# Comparative genomics of the white-rot fungi, *Phanerochaete carnosa* and *P. chrysosporium*, to elucidate the genetic basis of the distinct wood types they colonize

**DOI:** 10.1186/1471-2164-13-444

**Published:** 2012-09-02

**Authors:** Hitoshi Suzuki, Jacqueline MacDonald, Khajamohiddin Syed, Asaf Salamov, Chiaki Hori, Andrea Aerts, Bernard Henrissat, Ad Wiebenga, Patricia A vanKuyk, Kerrie Barry, Erika Lindquist, Kurt LaButti, Alla Lapidus, Susan Lucas, Pedro Coutinho, Yunchen Gong, Masahiro Samejima, Radhakrishnan Mahadevan, Mamdouh Abou-Zaid, Ronald P de Vries, Kiyohiko Igarashi, Jagjit S Yadav, Igor V Grigoriev, Emma R Master

**Affiliations:** 1 Department of Chemical Engineering & Applied Chemistry, University of Toronto, 200 College Street, Toronto, ON, M5S 3E5, Canada; 2 Environmental Genetics and Molecular Toxicology Division, Department of Environmental Health, University of Cincinnati College of Medicine, Cincinnati, OH, 45267-0056, USA; 3 US Department of Energy Joint Genome Institute, 2800 Mitchell Dr., Walnut Creek, California, 94598, USA; 4 Architecture et Fonction des Macromolécules Biologiques, Aix-Marseille Université, CNRS, UMR 6098, 163 Avenue de Luminy, 13288, Marseille, France; 5 Department of Biomaterials Sciences, Graduate School of Agricultural and Life Sciences, University of Tokyo, l-l-l, Yayoi, Bunkyo-ku, Tokyo, 113-8657, Japan; 6 Great Lakes Forestry Centre, 1219 Queen Street East, Sault Ste. Marie, Ontario, Canada, P6A 2E5; 7 Centre for the Analysis of Genome Evolution and Function, University of Toronto, 25 Willcocks Street, Toronto, ON, Canada, M5S 3B3; 8 CBS-KNAW Fungal Biodiversity Centre, Uppsalalaan 8, 3584, CT, Utrecht, The Netherlands

**Keywords:** *Phanerochaete carnosa*, Comparative genomics, *Phanerochaete chrysosporium*, Softwood degradation

## Abstract

**Background:**

Softwood is the predominant form of land plant biomass in the Northern hemisphere, and is among the most recalcitrant biomass resources to bioprocess technologies. The white rot fungus, *Phanerochaete carnosa*, has been isolated almost exclusively from softwoods, while most other known white-rot species, including *Phanerochaete chrysosporium*, were mainly isolated from hardwoods. Accordingly, it is anticipated that *P. carnosa* encodes a distinct set of enzymes and proteins that promote softwood decomposition. To elucidate the genetic basis of softwood bioconversion by a white-rot fungus, the present study reports the *P. carnosa* genome sequence and its comparative analysis with the previously reported *P. chrysosporium* genome.

**Results:**

*P. carnosa* encodes a complete set of lignocellulose-active enzymes. Comparative genomic analysis revealed that *P. carnosa* is enriched with genes encoding manganese peroxidase, and that the most divergent glycoside hydrolase families were predicted to encode hemicellulases and glycoprotein degrading enzymes. Most remarkably, *P. carnosa* possesses one of the largest P450 contingents (266 P450s) among the sequenced and annotated wood-rotting basidiomycetes, nearly double that of *P. chrysosporium*. Along with metabolic pathway modeling, comparative growth studies on model compounds and chemical analyses of decomposed wood components showed greater tolerance of *P. carnosa* to various substrates including coniferous heartwood.

**Conclusions:**

The *P. carnosa* genome is enriched with genes that encode P450 monooxygenases that can participate in extractives degradation, and manganese peroxidases involved in lignin degradation. The significant expansion of P450s in *P. carnosa*, along with differences in carbohydrate- and lignin-degrading enzymes, could be correlated to the utilization of heartwood and sapwood preparations from both coniferous and hardwood species.

## Background

The lignocellulose fraction of plant cell walls is the most abundant renewable carbon source on earth, and is a key resource for substituting petroleum in the production of energy, chemicals and materials. Among the various types of lignocellulosic biomasses, softwood is the predominant form of land plant biomass in the Northern hemisphere [1]. Softwood (coniferous) and hardwood (deciduous) fiber mainly differ in the structure and composition of hemicellulose and lignin components. For instance, glucuronoxylan comprises approximately 20–30% of the secondary cell wall polysaccharides in hardwood, while galactoglucomannan is the main hemicellulose in secondary cell walls of softwood. Moreover, softwoods contain mainly guaiacyl lignin, whereas hardwoods contain varying ratios of syringyl and guaiacyl lignins [2]. The recalcitrance of softwood lignocellulose to bioprocess technologies has been attributed to its higher lignin content, smaller pore size, and fewer hemicellulose-derived acetyl groups in comparison with hardwood [3].

Although relatively few organisms are known to effectively utilize all components of lignocellulose, white-rot basidiomycetes transform cellulose, hemicellulose and lignin, and so are regarded as essential contributors to global carbon cycling [4]. While most known white-rot species, including the model fungus *Phanerochaete chrysosporium*[5,6], have been mainly isolated from hardwoods, the white-rot basidiomycete, *Phanerochaete carnosa*, has been isolated almost exclusively from softwoods, including *Abies balsamea* (balsam fir), *Abies concolor* (white fir), and *Pinus ponderosa* (ponderosa pine) [7]. Previous studies have revealed differences in gene expression between *P. carnosa* and *P. chrysosporium*, suggesting that *P. carnosa* may possess an enzyme complement that is efficient for softwood bioconversion. For example, proteomic characterization of *P. carnosa* secretomes showed that *P. carnosa* produces a glycoside hydrolase (GH) family 2 mannanase, a multicopper oxidase, and glycopeptides that likely participate in carbohydrate and lignin degradation [8], which were not previously identified in proteomic analysis of *P. chrysosporium*[6,9]. More recent transcriptomic analyses evaluated gene expression in *P. carnosa* grown on various softwood species (white spruce, lodgepole pine, and balsam fir), as well as a hardwood (sugar maple). Notably, transcripts predicted to encode lignin-degrading activities (particularly manganese peroxidases) were more abundant than those predicted to encode carbohydrate-active enzymes [10], which is in contrast to earlier studies of *P. chrysosporium* grown on wood that revealed comparatively high levels of transcripts encoding carbohydrate-active enzymes [11].

Key requirements for the biotransformation of particular biomass resources could be elucidated through comparative analysis of closely related lignocellulose-degrading fungi having different substrate preferences. For instance, genomic comparison of *P. chrysosporium* and the softwood-degrading, model brown-rot fungus, *Postia placenta*, revealed that brown-rot is characterized by the contraction of multiple gene families, including cellobiohydrolases and cellulose-binding domains [12]. Moreover, comparative analysis of *Aspergillus* genomes identified correlations between genome content, plant polysaccharide degradation, and respective biotope [13]. Given the apparent differences in substrate preference of *P. carnosa* and *P. chrysosporium*, and their phylogenetic similarity based on internal transcribed spacer (ITS) region sequences [14], it is anticipated that comparative analysis of *P. carnosa* and *P. chrysosporium* genomes could reveal enzymes and metabolic pathways that are key to efficient biotransformation of recalcitrant softwood feedstocks. Accordingly, the present study reports the first analysis of the *P. carnosa* draft genome, and compares *P. carnosa* and *P. chrysosporium* in terms of genome composition and organization, as well as growth on model and woody substrates. These analyses revealed significant expansion of P450 genes in *P. carnosa* compared to *P. chrysosporium*, and highlighted several differences in carbohydrate active enzymes and lignin-degrading enzymes, which may facilitate softwood utilization by *P. carnosa*.

## Results and discussion

### Assembly and overview of the *P. carnosa* genome

The net length of the *P. carnosa* genome sequence was 46.3 Mb, and while distributed over 1137 scaffolds, the six largest scaffolds contained half of the total sequence and 57% of the 13,937 predicted genes ( Additional file 1: Table S1, Table S2, Table S3). A summary of functional annotations by several classifications is shown in Additional file 1: Table S4. Of these genes, those predicted to encode P450 monooxygenases, MFS transporters, and signaling proteins (e.g. WD40 and protein kinases), comprised the largest families based on PFAM domains of translated sequences, and were expanded relative to other Basidiomycetes ( Additional file 1: Table S5). Notably, the *P. carnosa* genome has 503 tandem gene duplication regions containing a total of 1660 genes, significantly higher numbers than the *P. chrysosporium* genome, which contains 305 duplication regions with 865 genes. Indeed, most of the top 50 PFAM domains are expanded in *P. carnosa* relative to *P. chrysosporium* ( Additional file 1: Table S5) and these are largely responsible for the differences in gene count and genome size between the two organisms*.*

**Figure 1:**
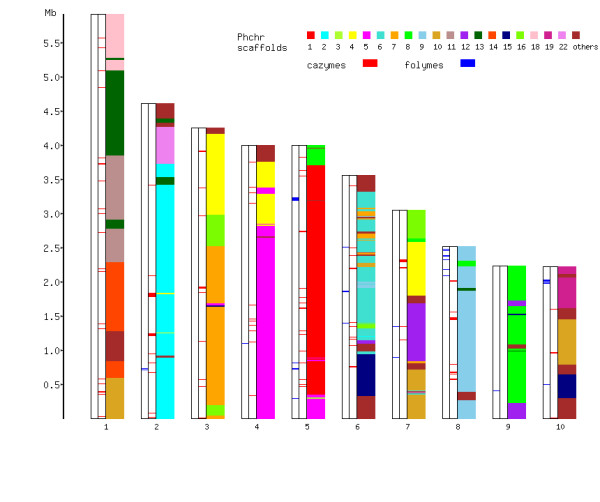
**Syntenic regions of the 10 largest scaffolds of* P. carnosa *with scaffolds of* P. chrysosporium *.** Scaffolds are shown as vertical bars, sequence starting from bottom to top, where the length of scaffolds is proportional to sequence length (largest: scaffold_1; smallest: scaffolds 9 and 10). Colors on the scaffolds refer to positions of genes that are the best homologs (orthologs) to corresponding *P. chrysosporium* scaffolds of the same color. Distribution of genes encoding CAZymes (red bars) and FOLymes (blue bars) across the scaffolds are also shown to the left of each scaffold.

A synteny display based on orthologous proteins in *P. carnosa* and *P. chrysosporium* revealed significant rearrangement in the evolutionary history of these two *Phanerochaete* genomes (Figure1). Most of the carbohydrate- active enzymes [15] were distributed across the ten largest scaffolds of the *P. carnosa* genome sequence. While the glycoside hydrolase (GH) families GH7 and GH3 were enriched on scaffold_2, and the GH5 family was enriched on scaffold_4, most carbohydrate-active enzymes (CAZymes) were only loosely clustered. By contrast, six of the seven predicted manganese peroxidases (MnPs) were located on scaffold_5, and three of the four predicted lignin peroxidases (LiPs) were located on scaffold_10 ( Additional file 2: Table S6), indicating that cluster formation of MnPs and LiPs is much tighter than that of CAZymes.

A primary aim of the current analysis was to compare the genomic content of *P. carnosa* and *P. chrysosporium*, and to correlate differences with their abilities to transform various compounds, including lignocellulosic substrates. Many previous reports demonstrate good correlation between genomic content and relative growth patterns on various carbon sources [13,16-18]. In those experiments, relative growth of a particular fungal species is determined by comparing the radius and density of the mycelia on a particular carbon source to that on D-glucose [19]. Relative growth patterns can then be compared between fungi, since differences in colony morphology and general differences in growth rates, are taken into account [16]. Accordingly, in addition to obtaining and analyzing the *P. carnosa* genome sequence, *P. carnosa* and *P. chrysosporium* were each grown in duplicate on 35 model carbon sources to determine how well respective genome contents correlated to relative growth measurements. Similar to previous analyses, relative growth of each fungus was determined separately by comparing the radius and density of mycelia on a particular carbon source to that on D-glucose; relative growth profiles were then compared between the two species ( Additional file 3: Figure S1) [19]. Notably, the relative growth of *P. carnosa* on casein was higher than that observed for *P. chrysosporium* ( Additional file 3: Figure S1), which is consistent with the considerable expansion of proteases encoded by *P. carnosa* ( Additional file 1: Table S5). Similarly, where possible in the following sections, the growth profiles of *P. carnosa* and *P. chrysosporium* are correlated to relative occurrence of CAZymes and fungal oxidative lignin enzymes (FOLymes) encoded by these white-rot fungi.

### Carbohydrate active enzymes (CAZymes) and correlation to carbon catabolism

Gene models encoding enzymes that were predicted to degrade, modify, or synthesize glycosidic bonds were annotated based on the CAZy classification scheme ( Additional file 4: Table S7) [20-22]. The number of predicted CAZymes encoded by *P. carnosaP. chrysosporium* and other Agaricomycotina genomes were also compared ( Additional file 1: Table S8). As is usually found among wood decaying fungi, *P. carnosa* and *P. chrysosporium* encode a large repertoire of GH61 members (11 and 13 GH61s, respectively). Whereas the *P. chrysosporium* genome encodes two GH61 proteins that are located together on scaffold_10 (Phchr41563 and Phchr41650, where numbers indicate protein IDs from the JGI database), *P. carnosa* encodes a single ortholog (Phaca263097), which was highly expressed during growth on different wood samples ( Additional file 3: Figure S2).

**Figure 2:**
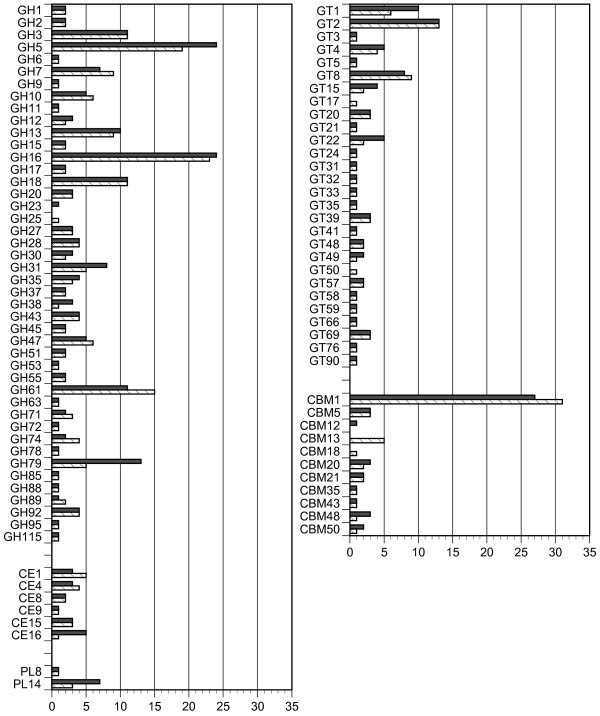
**Distribution of CAZymes in* P. carnosa *(shadowed) and* P. chrysosporium *(lined).**

**Figure 3:**
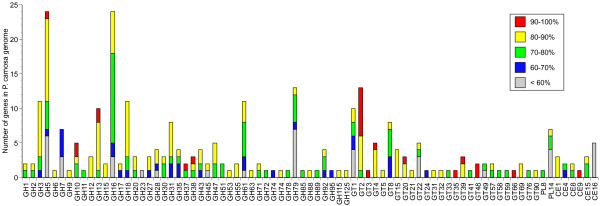
**The distribution of percent identities of CAZyme members between* P. carnosa *and* P. chrysosporium *.** For each query sequence (* P. carnosa * genes), the highest percent identity in blastp was scored. The numbers of * P. carnosa * genes are indicated according to the percent identities.

Although similar distributions of broad CAZy classifications were observed, closer inspection of particular CAZy families revealed notable differences between *P. carnosa* and *P. chrysoporium* (Figure2). For instance, both families GH23 and GH25 encode lysozyme activity, and the absence of GH25 in *P. carnosa* appeared to be compensated by a gain of a GH23 enzyme. Likewise, the absence of GT50 (α-1,4-mannosyltransferase) enzymes and CBM18s in *P. carnosa* was balanced by a gain in genes that likely encode functionally similar proteins from families GT22 and CBM12. After family 1 CBMs, CBM13s comprise the largest CBM family encoded by *P. chrysosporium* whereas *P. carnosa* totally lacks members from this CBM family. Although sugar binding by *P. chrysosporium* CBM13s has not been characterized, xylooligosaccharides were shown to associate with a CBM13 binding domain from *Streptomyces lividans*[23]. Families GH5, GH79, CE16, and PL14 appear to have expanded in *P. carnosa* relative to *P. chrysosporium* (Figure2), and contained a comparatively high fraction of proteins with relatively low identity (<60%) to orthologous proteins in *P. chrysosporium* (Figure3). While families GH5 and CE16 include enzymes involved in hemicellulose degradation, GH79 includes enzymes involved in processing glycoproteins, including those found in plant cell walls [24].

Given the expansion of GH5 members in *P. carnosa* and *P. chrysosporium* (24 and 19 GH5s, respectively), along with the role of GH5 enzymes in degrading (galacto)glucomannan present in softwood, and proficient colony development of *P. carnosa* on guar gum (galactomannan) compared to birchwood xylan and pectin ( Additional file 3: Figure S1), a phylogenetic analysis of GH5 protein sequences was performed to determine whether *P. carnosa* encodes new branches within this enzyme family ( Additional file 3: Figure S3). This analysis identified one ortholog of *P. chrysosporium* β-mannanases (Phchr5115 and Phchr140501; Man5C and Man5D, respectively) in *P. carnosa* (Phaca248589), and revealed that Phaca168586 clusters on a neighboring branch and with an orthologous protein most similar to mannanases. Three additional GH5 sequences (Phaca253799, Phaca194783 and Phaca194761) formed a branch in the protein phylogeny that lacked representation from *P. chrysosporium*. Since Phaca168586 shares comparatively low identity (56%) with the closest homolog in *P. chrysosporium* ( Additional file 1: Table S9), and Phaca253799 was the only gene product from the unique phylogenetic branch that was upregulated in *P. carnosa* during growth on wood [10], Phaca168586 and Phaca253799 might be particularly interesting to target for future biochemical characterization.

### Sugar transport and intracellular metabolism

In addition to the secretion of extracellular glycoside hydrolases, the ability to metabolize wood polysaccharides depends on the transport of resulting sugars and oligosaccharides, as well as intracellular conversion of these compounds.

Although genes encoding major facilitator superfamily (MFS) transporters appeared to have expanded in the *P. carnosa* genome, a phylogenetic analysis of predicted sugar transporters encoded by *P. carnosaP. chrysosporium*, and other fungi formed eight sequence clusters, and revealed a similar distribution of *P. carnosa* and *P. chrysosporium* proteins ( Additional file 3: Figure S4). A previous transcriptomic analysis of *P. carnosa* revealed comparatively high expression of Phaca263731 on nutrient rich medium [10], while Phchr137220, which also clusters in Group III, is upregulated in cellulose cultivations compared to glucose cultivations [25]. More interestingly, genes encoding four of the five Group VIII transport proteins were upregulated in *P. carnosa* grown on wood substrates ( Additional file 3: Figure S4; [10]). This group comprises predicted cellobiose transporters previously found in yeasts and filamentous fungi [26-29]. Notably, a Group VIII cellodextrin transporter from *N. crassa* (NcNCU00801 and 08114) has been biochemically characterized [30], supporting that *P. carnosa* transporter proteins found in this group could have a role in cellobiose and/or cellodextrin uptake.

Sugar metabolism by *P. carnosa* and *P. chrysosporium* was further investigated by using corresponding genome sequences to reconstruct an entire metabolic network for each organism [31]. Gene products predicted to participate in central metabolism were then manually curated and expression levels were assigned from previous transcriptomic analyses ( Additional file 5: Table S10). The results of this analysis revealed that the pentose catabolic pathway and the galacturonic acid pathway were induced in *P. carnosa* during growth on lignocellulosic substrates compared to nutrient medium, and that this response was accompanied by an increase in the expression of a transketolase, which catalyzes the conversion of D-xylulose-5-P to erythrose-4-phosphate in the pentose phosphate pathway. Enzymes that catalyze the first step in rhamnose and galactose metabolism were also induced in *P. carnosa* wood cultivations [10]. Similar trends were observed in *P. chrysosporium*, although it has fewer gene products in the corresponding metabolic pathways ( Additional file 5: Table S10).

Notably, growth studies revealed that *P. carnosa* grew poorly on birchwood xylan and pectin ( Additional file 3: Figure S1). Since the pentose and the galacturonic acid catabolic pathways likely promote the metabolism of pectin and xylan-derived sugars, metabolic models predicted herein suggest that poor growth of *P. carnosa* on pectin and xylan substrates could result from low expression of extracellular pectinase and xylanase enzymes or sugar transporters, rather than intracellular metabolism of hydrolysis products.

### Enzymes for lignin degradation, and conversion of related aromatic compounds

The most evident difference in lignin-degrading activities encoded by *P. carnosa* and *P. chrysosporium* was the distribution of predicted manganese peroxidases (MnP) and lignin peroxidases (LiP) ( Additional file 1: Table S11) [32]. The *P. carnosa* genome encodes eleven Class II peroxidases: seven manganese peroxidases (MnP: Phaca256980, 144982, 256984, 256991, 256997, 94399, 262882) and four lignin peroxidases (LiP: Phaca212237, 263501, 213241, 152156). By comparison, *P. chrysosporium* encodes five MnPs and ten LiPs ( Additional file 1: Table S11), suggesting that the two species might primarily rely on different peroxidase families for lignin decay. Growth profiling demonstrated that while both fungi grew well on wheat bran containing approx. 3% lignin, *P. chrysosporium* grew better than *P. carnosa* on cotton seed pulp (approx. 30% lignin) ( Additional file 3: Figure S1), suggesting a higher ligninolytic ability for *P. chrysosporium*. This result is consistent with the expansion of MnPs and reduction of LiPs in *P. carnosa* compared to *P. chrysosporium*, and the typically higher redox potential of LiP oxidoreductases.

A phylogenetic tree was previously constructed to infer the relationship of Class II peroxidases from *P. carnosa* and *P. chrysosporium*, and tree topology indicated gene expansion after *P. chrysosporium* and *P. carnosa* speciation [10]. Closer analysis of the protein phylogeny suggested that a common ancestor of *P. carnosa* and *P. chrysosporium* likely encoded three *mnp* genes and at least three *lip* genes. An analysis of intron distribution revealed the existence of up to seven conserved intron positions in the coding regions of *P. carnosa* and *P. chrysosporium mnp* genes, and up to ten conserved intron positions in the coding regions of the *lip*s ( Additional file 3: Figure S5). Most of these genes have lost one or two introns, suggesting a role for retrotransposition in gene expansion.

Among the nine predicted multi-copper oxidases (MCO) was a conventional ferroxidase (*fet3*; Phaca141262), and seven genes with high similarity (> 65% amino acid identity) to *P. chrysosporium mco4* (Phchr10581): these included Phaca261553, Phaca261563, Phaca149761, Phaca100787, Phaca100639, Phaca 149824, Phaca186926, which were all located on scaffold_8. Two additional genes, Phaca261609 and Phaca60261, were more closely related to *P. chrysosporium mco3* and *mco2*, respectively. Notably, only two of these MCO-encoding genes, Phaca141262 (*fet3*) and Phaca100639 (*mco4*), appear to be upregulated during growth on wood [10]. The predicted cellobiose dehydrogenase (*cdh*: Phaca259608) and an ortholog to *cir1* (cellulose-binding iron reductase, Phaca161126) were also up-regulated in *P. carnosa* during growth on wood [10]. As in the case of *P. chrysosporiumP. carnosa* does not appear to encode laccases *sensu stricto*. However, recent work with a *Phanerochaete flavido-alba* MCO shows that some members of the ferroxidase/laccase group may in fact have laccase activity [33].

Various enzymes are proposed to supply the H_2_O_2_ required for oxidase activity, the best established of which is glyoxal oxidase (GLOX, FOLy LDA3). The *P. carnosa* genome contains one *glox* gene (Phaca258261) and five related copper radical oxidases (*cro*: Phaca123913, Phaca259359, Phaca143144, Phaca263533, Phaca263528), compared to one *glox* and six related *cro* in *P. chrysosporium* ( Additional file 1: Table S12). Phaca123913, Phaca259359, and Phaca143144 were most closely related to *cro1cro2*, and *cro6* of *P. chrysosporium* (Phchr259359, Phchr123913, Phchr258261), respectively. Both Phaca263533 and Phaca263528 were similar to *cro3cro4*, and *cro5*, and contain repeated WSC domains of unknown function. These two WSC-containing *cro*s are found within the *lip* physical gene cluster on scaffold_10. Notably, of the five copper radical oxidases identified here, only Phaca263528 was upregulated in *P. carnosa* during growth on wood [10].

### Cytochrome P450 monooxygenases

Fungi encode a large repertoire of P450 genes [5,12,34], and in wood-rotting basidiomycetes in particular, P450s are known to be involved in the oxidation of phenolic and non-phenolic aromatic compounds with substructures similar to those occurring in lignin and extractive compounds that otherwise inhibit microbial growth [35-38]. Accordingly, the distinct lignin composition of hardwoods and softwoods, as well as the variety of extractives produced by different wood species, provoked an interest to compare *P. carnosa* and *P. chrysosporium* in terms of the P450s they encode.

The current genomic analysis revealed that *P. carnosa* possesses one of the largest P450 contingents (266 P450s) among the sequenced and annotated wood-rotting basidiomycetes, much higher than that of *P. chrysosporium* (149 P450s) [34], somewhat larger than that of the brown rot fungus *P. placenta* (236 P450s) [12], and higher than any of the 31 fungal genomes recently reported by Floudas et al. [18]. Among the large number of tandem gene duplication events in the *P. carnosa* genome, the largest tandemly duplicated gene family is P450 with 38 duplication regions resulting in 80 genes, while corresponding numbers in the *P. chrysosporium* genome are 15 and 23 ( Additional file 1: Table S13). Roughly 33% of the P450 genes encoded by *P. carnosa* appear to be tandemly duplicated. According to the phylogenetic analysis, tandem duplication of at least 15 P450 genes occurred after divergence from *P. chrysosporium*. For example, four tandemly duplicated genes found in scaffold 7 (1419983–1433634) were 80-90 identical, and shared roughly 60% identity with the best homolog in *P. chrysosporium*.

**Figure 4:**
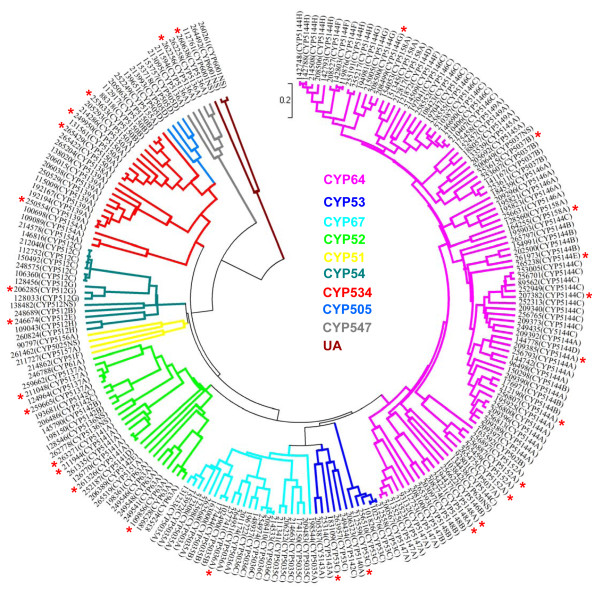
**Phylogenetic tree of* P. carnosa *cytochrome P450 proteins (P450ome).** Phylogenetic analyses were conducted in MEGA4 using the UPGMA method [75]. A total of 210 P450s (≥ 330 amino acids length) were grouped under ten existing clans and one unassigned clan (UA), with clans shown in different colors. P450 members that were upregulated in wood degrading cultures [10] are indicated with asterisks and are listed in Additional file 1: Table S15.

**Figure 5:**
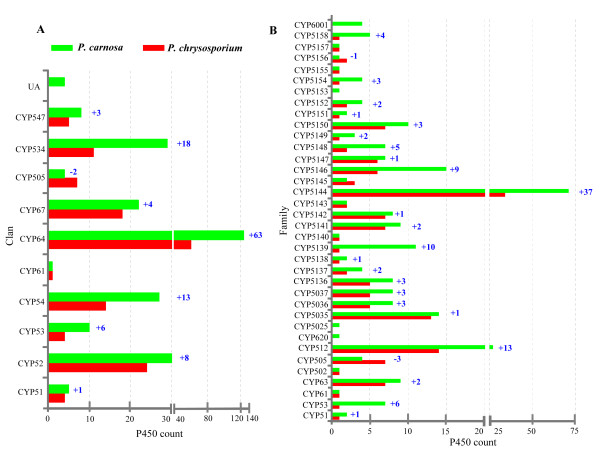
**Comparative evolutionary analysis of the P450omes of* P. carnosa *and* P. chrysosporium *.** The two P450omes were compared at the clan (**A**) and family (**B**) levels. In the * P. carnosa * P450ome (266 P450s) (green bars), 10 of the 11 clans and 32 of the 36 families corresponded to those present in * P. chrysosporium * (149 P450s) (red bars). Numbers on the * P. carnosa * clan or family bars represent the membership expansion (+) or reduction (−) in that clan or family as compared to * P. chrysosporium *.

The 266 P450s of *P. carnosa* were grouped into 10 known P450 clans, 36 families and 77 subfamilies, with CYP64 being the largest clan with 124 members (Figure4Additional file 1: Table S14). In comparison to *P. chrysosporium*, several P450 clans (CYP52, CYP53, CYP54, CYP64, CYP67, CYP534 and CYP547) and many families had expanded in *P. carnosa* (Figure5A and B). Interestingly, CYP64 and CYP52 were nearly doubled in *P. carnosa* and genes from these clans represented over half of the 28 unique P450 gene transcripts that were upregulated in *P. carnosa* wood degrading cultures ([10], Additional file 1: Table S15).

### Transformation of softwood and hardwood components by *P. carnosa* and *P. chrysosporium*

The genomic analysis of *P. carnosa* revealed that among the most remarkable differences between this organism and *P. chrysosporium* was the apparent enrichment in P450 genes and the difference in distribution of LiP and MnP genes. *P. carnosa* has been mainly isolated from softwood wood species, and so an interesting prospect is that growth of this organism on softwood is facilitated by the oxidative transformation of guaiacyl lignin and otherwise inhibitory wood extractives. To test this possibility, *P. carnosa* and *P. chrysosporium* were grown on heartwood and sapwood preparations from various hardwoods (sugar maple, yellow birch, trembling aspen) and softwoods (red spruce, white spruce, balsam fir, red pine), and residual wood extractives and lignin were measured using Ultra High Performance Liquid Chromatography (UPLC) and Fourier transform infrared spectroscopy (FT-IR), respectively.

**Figure 6:**
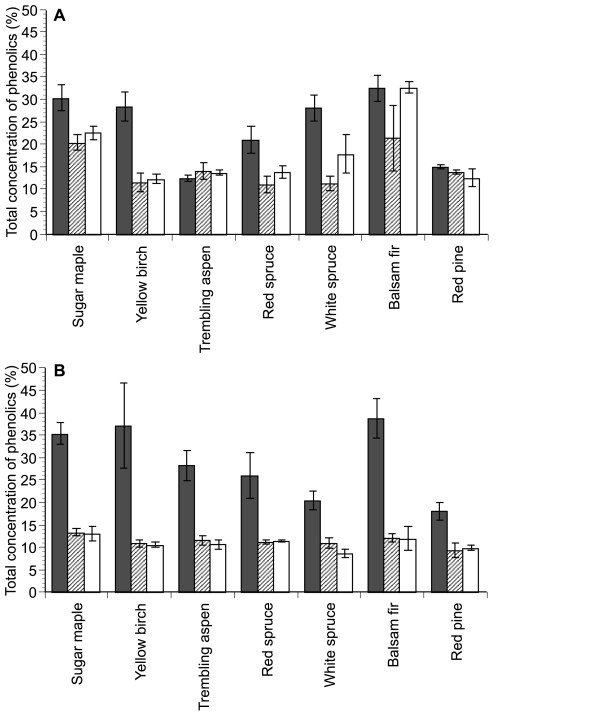
**Concentration of phenolic compounds in whole extractives of wood samples.*** P. carnosa * (lines) and * P. chrysosporium * (white) were grown on heartwood (**A**) and sapwood (**B**) wood samples at 27°C. * P. carnosa * grew significantly slower than *P. chrysosporium*, and so samples were taken for analysis after 42 and 18 days of cultivation for * P. carnosa * and * P. chrysosporium*, respectively (for colony diameter, see Additional file 3: Figure S6). Grey bars indicate the phenolic content of wood samples prior to fungal cultivation. The amount of phenolics in total extractives was measured using the F-C reagent method and calculated as percent concentration [77]. Gallic acid was used to generate a calibration curve. Error bars show the standard deviation in biological triplicates.

UPLC analyses revealed that while *P. carnosa* and *P. chrysosporium* similarly reduced the total phenolic content of sapwood samples, *P. carnosa* transformed a higher fraction of phenolics in most of the heartwood samples, including those from softwood species containing high initial phenolic content (Figure6). Interestingly, *P. chrysosporium* transformed a broader range of heartwood phenolics in maple than *P. carnosa*, and this trend was also observed albeit to a lower extent, for aspen heartwood ( Additional file 1: Table S16). Moreover, growth of *P. chrysosporium* on heartwood from softwood species was only observed in red spruce cultivations ( Additional file 3: Figure S6), which contained relatively low initial phenolic content. In some cases, the transformation of specific phenolic compounds by *P. carnosa* could also be correlated to growth. In particular, the transformation of kaempferol 3-O-β-D-glucoside and naringin in heartwood from white spruce was higher for *P. carnosa* than *P. chrysosporium* ( Additional file 1: Table S16), and *P. carnosa* exhibited better growth on white spruce ( Additional file 3: Figure S6). Both kaempferol 3-O-β-D-glucoside and naringin are sugar-containing phenolic compounds, where the sugar moiety can be released by GH1 β-glucosidase activity and GH13, GH15 or GH78 α-rhamnosidase activity, respectively. Since GH families 1, 13, 15 and 78 did not substantially differ between *P. carnosa* and *P. chrysosporium*, detoxification of the phenolic moiety of kaempferol 3-O-β-D-glucoside and naringin might have promoted the utilization of these compounds.

Aspen and pine wood samples were also analyzed by FT-IR to identify differences in lignin and carbohydrate components resulting from *P. carnosa* or *P. chrysosporium* cultivation. Principal component analysis (PCA) of wood FT-IR spectra in the fingerprint region (800–1800 cm^-1^) clearly separated wood samples decayed by *P. carnosa* and *P. chrysosporium* ( Additional file 3: Figure S7). Overall, wood samples collected from *P. carnosa* cultivations were distinguished from control samples by a reduction in lignin ( Additional file 3: Figure S7; Additional file 1: Table S17); wood samples from *P. chrysosporium* cultivations were further distinguished by more extensive polysaccharide degradation ( Additional file 3: Figure S7; Additional file 1: Table S17). Notably, peak intensities at wavenumbers characteristic of cellulose absorption were reduced in heartwood pine cultivations, even though growth of *P. chrysosporium* was not visible on this wood sample ( Additional file 3: Figure S6). The decay patterns revealed by the FT-IR analysis suggest that while *P. chrysosporium* is known to elicit simultaneous decay of lignin and polysaccharides, *P. carnosa* likely elicits sequential degradation of cell wall components. These results are consistent with previous reports that illustrate comparatively high expression of genes encoding lignin-degrading activities in *P. carnosa*, and cellulose-degrading activities in *P. chrysosporium*, during growth of these fungi on various wood samples [10,11,39].

## Conclusions

An underlying hypothesis of the current study was that identifying subtle differences in the distribution and sequence of genes encoded by *P. carnosa* and *P. chrysosporium* would facilitate the discovery of candidate enzymes that promote the conversion of recalcitrant softwood feedstocks. Indeed, the comparative genomic analysis of these related white-rot fungi revealed a tangible set of enzyme classes that could promote the conversion of softwood. In particular, this study revealed that the most divergent CAZymes belonged to families GH5, GH79, CE16, and PL14, that the relative abundance of genes encoding MnP and LiP oxidoreductases was reversed in *P. carnosa* compared to *P. chrysosporium*, and that *P. carnosa* encodes the largest contingent of P450 enzymes than any other basidiomycete characterized to date.

Specific hemicellulases, particularly from family GH5, were identified for future biochemical characterization. However, the clearest correlations between growth studies and differences in encoded enzymes were in regards to lignin removal and ability to transform wood extractives. While the predicted differences in the redox potential of MnP and LiP was consistent with slower growth of *P. carnosa* on wheat bran and cotton seed pulp compared to *P. chrysosporium*, oxidants produced by MnP might diffuse more easily through dense lignocellulose structures than LiP associated oxidants [40], *P. carnosa* removed a higher proportion of phenolic extractives in heartwood samples of softwood than did *P. chrysosporium*; *P. carnosa* also showed better growth on these feedstocks. Given the comparatively high expression of P450 genes from the CYP64 and CYP52 clans in *P. carnosa* grown on woody substrates [10], an intriguing possibility is that the expansion of CYP64 and CYP52 families discovered through genome sequencing enables *P. carnosa* to colonize heartwood from both softwood and hardwood species. The implication of this analysis is that detoxification of wood extractives and ability to degrade dense lignin structures may be key to enhancing softwood bioconversion.

## Methods

### Data access

*P. carnosa* genome assembly and annotations are available via JGI Genome Portal MycoCosm [41] and DDBJ/EMBL/GenBank under the accession AEHB00000000.

### Fungal strains

*Phanerochaete carnosa* strain HHB-10118-sp and *Phanerochaete chrysosporium* strain RP-78 were obtained from the U.S. Department of Agriculture (USDA) Forest Products Laboratory (Madison, WI). These strains were maintained on YMPG agar plates under stationary conditions at 27°C. YMPG medium contained 2 g yeast extract, 10 g malt extract, 2 g peptone, 10 g glucose, 2 g KH_2_PO_4_, 1 g MgSO_4_·7H_2_O, and 15 g agar per 1 L in H_2_O.

### Confirming Homokaryosis

Single-copy *cdh* and *fet3/ftr1* genes of *P. carnosa* were amplified by PCR and sequenced to reveal an absence of allelic polymorphism [42]. Specifically, PCR was performed using AccuPrime Pfx DNA polymerase and Reaction Mix (Invitrogen) with 160 ng of genomic DNA and 12.5 pmol of each primer in a 25 μL reaction volume. Primers for amplification of *cdh* (5'-TCKGARGCHGGVAAGAARGT-3' and 5'-GGVCCRATVCCGCTYTGGAA-3') were designed based on conserved sequences from six Basidiomycete fungi, and the PCR cycle was run as follows: 95°C for 9 min, 30 cycles of (95°C for 1 min, 50°C for 2 min, 72°C for 2 min), and 72°C for 15 min. Primers for amplification of *fet3*/*ftr1* (5'-TGGACGATCTGGAACTTGTG-3' and 5'-TCTCACGGAAGACGATGAAG-3') were based on the corresponding *P. chrysosporium* sequence, and the PCR cycle was run as follows: 95°C for 9 min, 30 cycles of (95°C for 1 min, 65°C for 2 min, 72°C for 3 min), and 72°C for 15 min. Amplified sequences were cloned into the pCR2.1-TOPO plasmid (Invitrogen) and sequenced at the Analytical Genetics Technology Centre (Toronto, ON, Canada) or the Centre for Applied Genomics (Toronto, ON, Canada). Absence of clamp connections normally produced by heterokaryons during cell division [43] was also confirmed by microscopic visualization of *P. carnosa* mycelia.

### Genome sequencing and assembly

The genome was sequenced using a combination of Sanger (4 kb, 8 kb, 40 kb paired end), 454 (Titanium unpaired; 3.7 kb, 5.2 kb, 6.1 kb, 14.5 kb Titanium paired end), and Illumina (3 lanes of 2x76 bp, 0.3 kb insert paired end) sequencing platforms ( Additional file 1: Table S1a). All general aspects of library construction and sequencing can be found at the JGI website [44]. The Illumina data was assembled with the Velvet assembler (version 0.7.55; [45]) with a hash length of 61 and the following options; -ins_length 250 -scaffolding no -exp_cov 16 -cov_cutoff 5, to produce an assembly with a final graph with 51765 nodes, n50 of 2413, max 22432, total 37740533, using 73178660/94020728 reads. Contigs greater than or equal to 800 bp in length were shredded into 1000 bp fragments with 800 bp overlap to be used by Newbler assembler. After eliminating possible contaminant data, the combined set of velvet fragments, 454 and Sanger reads was assembled with the Newbler assembler, version 2.5-internal-10Apr08-1 with the following options; -fe reads2remove.FQC -consed -nrm -finish -info -rip -sio -g -ml 31 -mi 98 -e 48, to a final estimated assembled coverage of 58X, 1137 scaffolds with an N/L50 of 6/3.6 Mb, and 2687 contigs with an L/N50 of 248/45.2 Kb ( Additional file 1: Table S1b). One round of automated gap closure using our in house gapResolution tool [46] resulted in a final assembly with 2272 contigs with an N/L50 of 139/74.8 kb. Newbler assembled consensus EST sequence data was used to assess the completeness of the final assembly using alignment with 90% identity and 85% coverage thresholds. This resulted in 90.33% placement.

### cDNA library construction, sequencing and assembly

Two clone cDNA libraries were constructed using RNA from *P. carnosa* mycelium grown in liquid YMPG medium and sequenced as described previously [47], with the minor difference that there were the two size ranges of cDNA (0.6 k-2 kb and >2 kb). The smaller cDNA insert library resulted in 9,971 ESTs for further processing and the larger insert library resulted in 9,530 ESTs. RNA purified from mycelia grown on mixed softwood was used to construct a 454 cDNA library according to the cDNA Rapid Library Preparation Method Manual (Rosch, Germany), yielding 1,139,862 ESTs for clustering and assembly. The entire set of 1,159,363 reads were assembled using Newbler (v2.3-PreRelease-6/30/2009) with default parameters resulting in 16,234 contigs greater than 50 bp. and 59,361 singlets.

### Genome annotation

The *P. carnosa* genome was annotated using the JGI annotation pipeline, which takes multiple inputs (scaffolds, ESTs, and known genes) and runs several analytical tools for gene prediction and annotation. Results were deposited in the JGI Genome Portal [48], a part of the integrated fungal resource MycoCosm [42] for further analysis and manual curation.

Genomic assembly scaffolds were masked using RepeatMasker [49] and the RepBase library of 234 fungal repeats [50]. tRNAs were predicted using tRNAscan-SE [51]. Using the repeat-masked assembly, several gene prediction programs falling into three general categories were used: 1) *ab initio* - FGENESH [52]; GeneMark [53], 2) *homology-based* - FGENESH+; Genewise [54] seeded by BLASTx alignments against GenBank’s database of non-redundant proteins (NR: [55]), and 3) *EST-based* - EST_map [56] seeded by EST contigs. Genewise models were extended where possible using scaffold data to find start and stop codons. EST BLAST alignments [57] were used to extend, verify, and complete the predicted gene models. The resulting set of models was then filtered for the best models, based on EST and homology support, to produce a non-redundant representative set of 13,937 gene models with characteristics described in Additional file 1: Table S2. This representative set was subject to further analysis and manual curation.

Predicted gene models were functionally annotated using SignalP [58], TMHMM [59], InterProScan [60], and BLASTp [61] against the National Center for Biotechnology Information nr database, and hardware-accelerated double-affine Smith-Waterman alignments [62] against [63] KEGG [64] and KOG [65]. KEGG hits were used to assign EC numbers [66], and Interpro and SwissProt hits were used to map GO terms [67]. Functional annotations of the representative set of genes are summarized in Additional file 1: Table S3 and Table S4. Multigene families were predicted with the Markov clustering algorithm (MCL [68]) using BLASTp alignment scores between proteins as a similarity metric.

### Carbohydrate active enzymes

All *P. carnosa* protein models were subjected to a procedure combining BLAST and HMMer3 searches against sequence libraries and HMM profiles derived from the families of glycoside hydrolases, polysaccharide lyases, carbohydrate esterases, glycosyltransferases and carbohydrate-binding modules featured in the CAZy database [15,69]. The models corresponding to glycoside hydrolase families GH1, GH2, GH3, GH5, GH6, GH7, GH9, GH10, GH11, GH12, GH16, GH28, GH31, GH43, GH45, GH51, GH53, GH55, GH61, GH74, GH79, GH115, carbohydrate esterase families CE1, CE15, CE16, carbohydrate-binding module family CBM1, as well as cellobiose dehydrogenase and cellulose-binding cytochrome *b*_562_[70] were manually checked using TBLASTN against the *P. carnosa* assembly database [48]. Gene models used for BLAST were obtained from the *P. chrysosporium* genome database [6,71]. In the case of multi-domain proteins, sequences encoding CBM and its associated domains were separately used as separate queries. TBLASTN programs were performed with an expectation value of 1.0E^-1^, and all other settings at default values.

### Sugar transporters

Protein models that were annotated as predicted sugar transporters and/or permeases in the *P. carnosa* genome portal v1.0 were used as queries for BLASTp against the NCBI protein database [55] and the *P. chrysosporium* genome portal version 2 to confirm these annotations. Annotations were considered accurate when either BLAST search gave an alignment to a predicted protein with an E-value of < 1e-10 and a score of ≥ 200. The *P. carnosa* genome was then re-searched using the *P. chrysosporium* genes selected above as queries to confirm that all of the gene models relevant to this analysis were selected.

For the phylogenetic analysis, multiple alignment was performed using MAFFT version 6 software [72] with the E-INS-i algorithm. The phylogenetic tree was then constructed from the multiple alignment using the bootstrapped neighbor-joining method (1000 bootstraps), and drawn using FigTree version 1.3.1 [73]. This analysis included 28 gene models from *P. carnosa* and 25 gene models from *P. chrysosporium*, while partial gene fragments were removed.

### Oxidoreductases

The BLASTp algorithm available through the JGI Fungal Genomics Program website [41] was used with default settings to search Agaricomycotina gene catalogue proteins against reference proteins. Hits were then blasted against the NCBI database [55] with default settings, and aligned to the reference protein sequences using the tool at the Genestream Bioinformatics Resource server [74]. Sequences were annotated to the reference protein when the bests hits to NCBI represented sequences of interest and the alignment showed at least 30% amino acid identity to the reference protein. Reference proteins were chosen based on biochemical evidence supporting their identity, and correspond to the following Genbank accession numbers: LO1 (laccase) LAC2_PLEOS, LO2 (peroxidases) LIG8_PHACH, LO3 (cellobiose dehydrogenase) CDH_PHACH, LDA1 (aryl alcohol oxidase) AAC72747, LDA2 (vanillyl-alcohol oxidase) VAOX_PENSI, LDA3 (glyoxal oxidase) AAA33747, LDA4 (pyranose oxidase) P2OX_PHLGI, LDA5 (galactose oxidase) XP_959153, LDA6 (glucose oxidase) XP_002910108, LDA7 (benzoquinone reductase) AAD21025, LDA8 (alcohol oxidase) AAB57849, methanol oxidase ALOX_PICAN, quinone reductase AF465406.

### P450 monooxygenases

Initial determination of the putative cytochrome P450 gene models in *P. carnosa* was made by searching the JGI whole genome database for the term ‘P450’. The resulting putative sequences were subjected to BLAST analysis and searched for the presence of the conserved P450 signature domains namely, the oxygen-binding motif ‘EXXR’ and the heme-binding motif ‘CXG’. P450s that showed both the domains were considered authentic and were grouped into families and subfamilies based on the existing nomenclature criteria of > 40% amino acid homology for assigning a family and > 55% for a subfamily. The families were then grouped into P450 clans, a higher order level of nomenclature that represents a cluster of P450 families across species, grouped based on relationships that are beyond the family designations [57]. P450 superfamily nomenclature rules were followed for assigning the clan, family and sub-family classification as earlier applied for P450ome classification in the model white-rot fungus, *P. chrysosporium*[34]. P450s that did not have *P. chrysosporium* P450 homologues were annotated based on the phylogenetic alignment with other P450s on the phylogenetic tree; the tree was constructed using Mega 4 *via* the bootstrap UPGMA method [75]. P450s showing both the conserved domains and a reasonable deduced protein length (≥ 330 aa) were used for the tree construction.

### Metabolic network reconstruction

The *P. carnosa* metabolic network was reconstructed using version 15 of the Pathway Tools Software from SRI International Inc [76]. This network was primarily constructed from the annotation using Enzyme Commission, Gene Ontology identifiers as well as name matching algorithms. The reconstructed metabolic network contained 1166 metabolites, 1630 enzymatic reactions and 11 transport reactions that were linked to 3292 enzymes. The completed network is available at [31].

### Cultivation on model and wood substrates

*P. carnosa* and *P. chrysosporium* were grown in duplicate on modified Kremer and Wood medium containing 1.5% agar with 35 different model carbon sources [19] for 13 and 3 days, respectively, after which pictures of the plates were taken to compare colony diameter and thickness. Relative growth was determined by comparing the radius and density of the mycelia on a particular carbon source to that on D-glucose; relative growth profiles were then compared between the two fungal species. The extent of growth relative to plates containing glucose are categorized from high to non-detectable using the following designations: +++, ++, +, ±, -. No differences were observed between duplicates on any of the substrates, so only one of the pictures is presented. Monomeric and oligomeric carbon sources were used at a final concentration of 25 mM, while pure polymers were used at a final concentration of 1%. Crude plant biomass was used at a final concentration of 3%. This procedure allows consistent, qualitative assessment of colony development on many substrates; it has been performed routinely for more than 100 fungal species and showed good overall correlations with the genome content of these species [16,18,19].

The following hardwood and softwood species were used for wood cultivations: sugar maple (*Acer saccharum*), yellow birch (*Betula alleghaniensis*), trembling aspen (*Populus tremuloides*), red spruce (*Picea rubens*), white spruce (*Picea glauca*), balsam fir (*Abies balsamea*), and red pine (*Pinus resinosa*). All wood samples were obtained from New Brunswick, Canada. A 50 cm bolt at 80 cm and 130 cm trunk heights were cut from each wood species. Sapwood and heartwood sections were then separated, air-dried, and processed separately using a Wiley mill (Thomas scientific, NJ, USA). Resultant wood powder was sifted using 2 mm and 0.425 mm diameter mesh sieves and powder that passed through the 2 mm sieve but was retained by the 0.425 mm sieve was recovered. Four grams of wood powder were placed on top of 5 g vermiculite powder (< 1 mm dia.) in a glass petri dish measuring 9 cm in diameter; 20 mL of H_2_O was gently added to the dish, and the dish was then autoclaved for 20 min. A 0.5 cm dia. agar plug taken from the growing edge of *P. carnosa* or *P. chrysosporium* cultivated on YMPG agar plates was then transferred to the centre of each plate, and incubated at 27°C under stationary conditions. To maintain moisture content, 5 mL H_2_O was added to each plate every week during cultivation. Fungal growth was quantified by measuring the diameter of the fungal colony growing on each wood powder. Three replicate cultivations were prepared for each fungal and wood species.

### Extractives analysis

An accelerated solvent extraction method (DIONEX, Application Note 335) was used to isolate wood extractives. To obtain standard samples for baseline analysis, 2 g of non-treated heartwood and sapwood samples from each wood species were mixed with approximately 0.45 g of diatomaceous earth, and then transferred to an 11 mL cell; the headspace in the cell was then filled with sand. Extraction was performed as follows; preheat 0 min, heat 5 min, static 5 min, flush 90%, purge 60 sec, cycles 5, pressure 1000 PSI, temperature 100°C, solvent 70% MeOH and 30% H_2_O (vol/vol). To obtain extractives from fungal-treated and control samples, each sample was mixed with solvent (70% MeOH, 30% H_2_O (vol/vol)) in a 10:1 ratio (solvent(mL):sample weight (mg)) and incubated for 24 h on an orbital shaker at room temperature, and then filtered. Supernatant was collected and a second extraction was performed on the filtered wood samples. Supernatants from the first and second extractions were combined, concentrated using a rotary evaporator, and then dried using a nitrogen evaporator.

Extractives were analyzed using Ultra performance liquid chromatography (UPLC). UPLC analysis was performed using a Waters Acquity Ultra Performance Liquid Chromatography equipped with a computer and Masslynx software, a binary solvent manager, a sample manager and an autoscan photodiode array spectrophotometer detector (PDA eλ). The UPLC was equipped with an Acquity UPLC BEH C18, 1.7 μm, (2.1 x 50 mm i.d.) reverse-phase analytical column from Waters housing a Van Guard BEH C18, 1.7 μm pre-column. All samples were dissolved in 70% acetonitrile: 30% H_2_O and diluted to a concentration of 10 mg/mL or to a minimum of 0.25 mL. Standards included gallic acid, methyl gallate, quercetin and rutin; 0.5 μL of each standard and 3 μL of samples were analyzed using gradient elution as shown in Additional file 1: Table S18. Column and auto-sampler temperature were maintained at 25°C. Two fixed detection wavelengths (280 nm and 350 nm) were used to monitor the eluting peaks. Resolved peaks were scanned by the photodiode array detector from 240 to 460 nm.

The F-C reagent method was used to calculate total phenolic concentration [77]. A calibration curve was created using gallic acid at concentrations of 25 mg/L, 50 mg/L, 100 mg/L, 250 mg/L, and 500 mg/L in Milli-Q water. Briefly, 20 μL of sample, gallic acid standard or blank was transferred to a 2.0 mL cuvette; 1.58 mL of Milli-Q water and 100 μL of F-C Reagent were then added, mixed and incubated for 5 min. Subsequently, 300 μL of sodium carbonate was added, mixed and incubated for 2 h at room temperature. Absorbance was measured at 765 nm using a Beckman 800 series spectrophotometer.

### FT-IR spectroscopy

Heartwood and sapwood of trembling aspen and red pine samples were collected before and after cultivation with *P. carnosa* and *P. chrysosporium*, and powdered using the mini-beadbeater-16 (Biospec products, USA); corresponding uninoculated controls were similarly processed. Two milligrams of wood powder were mixed with KBr (200 mg) and the mixture was pelletized using a die (1.3 cm diameter) and a hydraulic press. A Bruker Tensor 27 FT-IR was used to record the absorbance between 4000 and 400 cm^-1^ with a resolution of 4 cm^-1^. Spectra representing the average of 32 scans were corrected for atmospheric vapor compensation; baseline was corrected using the rubber band method (Opus software, v. 5.0). Spectra were normalized for unit-vector and mean-centred prior to the principal component analysis (PCA) using Unscrambler v. 9.7 software.

## Competing interests

The authors declare that they have no competing interests.

## Authors’ contributions

HS participated in the manual gene annotation and phylogenetic analysis of CAZy enzymes and transporter proteins, fungal growth study and FT-IR analysis, and drafted the manuscript. IVG, AA, AS, KB, EL, KL, AL, and SL participated in the genome sequencing and annotation. JM confirmed homokaryosis of *P. carnosa*, prepared fungal DNA and RNA samples for sequencing and participated in sequence analysis of oxidoreductase enzymes. KS and JY participated in annotation and phylogenetic analysis of P450 enzymes. CH, KI and MS participated in manual annotation of CAZy enzyme genes. BH and PC performed computational searches and annotation of CAZy enzymes. AW, PAvK and RPdV participated in fungal growth profiling and manual analysis of carbohydrate metabolism. YG participated in sequence alignment. RM participated in reconstruction of metabolic network. MAZ performed wood component analysis. ERM conceived of the study, participated in its design and coordination, and helped to draft the manuscript. All authors read and approved the final manuscript.

## Supplementary Material

Additional file 1 **Table S1.***P. carnosa* sequencing summary. This describes the summary of libraries that were constructed for genome sequencing. **Table S2.***P. carnosa* assembly summary. This describes the summary of the genome sequence assembly. **Table S3.** Gene model support by different lines of evidence. Statistics of predicted gene models. **Table S4.** Summary of *P.carnosa* annotations. This summarizes genome annotation according to various classifications. **Table S5.** Top 50 PFAM domains in *Phanerochaete* genomes. List of top 50 PFAM domains annotated in *Phanerochaete* genomes. **Table S8.** Comparison of the number of CAZymes in wood decaying basidiomycotina. General comparison of CAZyme gene members in various basidiomycotina [5,6,12,17,78,79]. **Table S9.** CAZymes that showed <60% identity to Phchr orthologs. List and gene expression data of CAZy members showing low sequence identity with orthologs in *P. chrysosporium*[10]. **Table S11.** Comparison of the number of FOLymes in *P. carnosa* and selected Agaricomycotina. General comparison of FOLy members between several Agricomycotina [78]. **Table S12.** Summary of oxidoreductases potentially involved in lignocellulose degradation by *P. carnosa* (Phaca) and *P. chrysosporium* (Phchr). Summary and comparison of specific oxidoreductase members between *Phanerochaete*s. **Table S13.** Tandem duplication of P450 genes in basidiomycete genomes. Summary table of P450 tandem duplication in known basidiomycete genomes [5,6,12,17,78-80]. **Table S14.** P450ome classification in *P. carnosa* and its membership comparison with *P. hrysosporium*. Summary table of P450 members in *P. carnosa* and *P. chrysosporium*. **Table S15.** P450s upregulated in wood degrading cultures. List of P450 members in *P. carnosa* that were upregulated during wood degrading cultivation [10]. **Table S16.** UPLC peaks corresponding to wood-derived phenolic compounds that were transformed by *P.carnosaor P*. *chrysosporium*. Peak annotation and analysis of UPLC chromatograms. **Table S17.** Assignment of FT-IR peaks decomposed by *P. carnosa* and *P. chrysosporium.* Summary of the peaks detected in FT-IR analysis (Figure S7) [81-83]. **Table S18.** Gradient method of UPLC elution. Summarizing elution method used in UPCL analyses. Click here for file

Additional file 2 **Table S6**. Genomic distribution of CAZymes and FOLymes on major scaffolds in *P. carnosa.* This table summarizes the distribution of CAZy and FOLy members located within the major scaffolds of the *P. carnosa* genome sequence. Click here for file

Additional file 3 **Figure S1.** Pictures of *P. carnosa* and *P. chrysosporium* grown on various carbon sources. *P. carnosa* grew significantly slower than *P. chrysosporium*; as a result, pictures were taken after 13 and 3 days of cultivation for *P. carnosa* and *P. chrysosporium*, respectively. Cultivations were performed in duplicate and no significant differences in colony diameter or thickness were observed between the duplicates on any of the carbon sources. Monomeric and oligomeric carbon sources were used at a final concentration of 25 mM, while pure polymers were used at a final concentration of 1%. Crude plant biomass was used at a final concentration of 3%. Relative growth was determined by comparing the radius and density of the mycelia on a particular carbon source to that on D-glucose. The extent of growth relative to plates containing glucose are summarized as follows, from high to non-detectable: +++, ++, +, ±, -. This semi-quantitative, consistent assessment of both colony diameter and thickness allowed comparisons to include a broad range of substrates, including those that yield too little mycelia for accurate weight measurement, or that could interfere with protein measurements or ergosterol production [84,85]. Complete growth profiles of *P. carnosa* and *P. chrysosporium*, and other fungi can be found at [19]. **Figure S2.** Phylogenetic tree of GH61 enzymes from *P. carnosa* and *P. chrysosporium*. Proteins are labeled with protein IDs from the JGI databases for *Phanerochaete carnosa* v1.0 (Phaca) and *Phanerochaete chrysosporium* v2.0 (Phchr). The sequences were aligned using MAFFT, and the tree was drawn by FigTree. In the heatmap bar, abbreviations are; Y, YMPG; F, balsam fir; P, lodgepole pine; S, white spruce; M, sugar maple [10]. Heat map represents the number of sequence reads per million kb as shown in this figure and described in [10], 0 (black) to 50,000 (pink). **Figure S3.** Phylogenetic tree of GH5 enzymes in *P. carnosa* and *P. chrysosporium*. The tree was generated as described in Figure S2. Of the 34 upregulated wood-degrading CAZymes, that were at least four times more abundant in *P. carnosa* grown on at least one wood substrate compared to nutrient medium, 5 (15%) were GH5 enzymes [10]. **Figure S4.** Phylogenetic tree of predicted sugar transporters and permeases from genomes of *P. carnosa* and *P. chrysosporium.* Proteins are labeled with protein IDs from the JGI database for *P. carnosa* v1.0 (Phaca) and *P. chrysosporium* v2.0 (Phchr). Protein sequences of transporters from other yeast and fungal species were used for pylogenetic comparison, including; An *Aspergillus nidulans*, Ao *Aspergillus oryzae*, Ca *Candida albicans*, Ci *Candida intermedia*, Gz *Gibberella zeae*, Hp *Hansenula polymorpha*, Kl *Kluyveromyces lactis*, Lb *Laccaria bicolor*, Nc *Neurospora crassa*, Pp *Postia placenta*, Ps *Pichia stipitis*, Sc *Saccharomyces cerevisiae*, Sp *Schizosaccharomyces pombe*, Tm *Tuber melanosporum*, and Tr *Trichoderma reesei*. The GenBank accession numbers of corresponding sequences are: AnHyp1 (XP_682442.1), AnHyp2 (XP_660070.1), AnMstA (CAC80843), Ao_BAE58341.1 (BAE58341.1), CaHgt1 (CAA76406), CaHgt4 (XP_723173), CaHgt11 (XP_719597), CiGxf1 (AJ937350), CiGxs1 (AJ875406), GzHyp1 (EAA74528), HpGcr1 (AAR88143), KlHgt1 (XP_451484), KlRag1 (XP_453656), KlRag4 (CAA75114), Lb_EDR07962 (EDR07962), NcHyp1 (XP_328858), NcHxt3 (CAD21508), NcNCU00801(EAA34565.1), NcNCU08114 (XP_963873.1), NcRco3 (CAE76420), Pp_115604 (EED81359), Ps_ABN65648.2 (ABN65648.2), PsSut1 (AAD00266), ScHxt1 (M82963), ScHxt7 (NP_010629), ScSnf3 (P10870), SpGht1 (Q9P3U6), Tm-CAZ81962.1 (CAZ81962.1), TrHxt1 (AAR23147), TrXlt1 (shown enlarged; AY818402), TrHxt2 (DQ852622; Ruohonen and Margolles-Clark, unpublished). The sequences were aligned using MAFFT, and the tree was drawn by FigTree. In the heatmap bar, abbreviations are: Y YMPG, F balsam fir, P lodge pole pine, S white spruce, M sugar maple [10]. Group I contains a predicted monosaccharide transporter (ID 100265) and hypothetical proteins with high similarity to known sugar transporters. Group IV also contains known glucose transporters found in yeast species and cellulolytic fungi, including *Trichoderma reesei* and *Neurospora crassa*. Group V consists of high affinity glucose transporters in yeast and hexose transporters in fungal species, including the xylose transporter found in *T. reesei*[86]. Group VI and VII contains predicted sugar transporters and putative sucrose transporters (ID 89844 and 254080). Group VIII consists of predicted cellobiose transporters found in yeasts and filamentous fungi, and biochemically characterized cellodextrin transporters from *N. crassa* (NcNCU00801 and 08114) [30]. **Figure S5.** Phylogeny, genome position, and intron distribution of genes encoding manganese peroxidases and lignin peroxidases. Protein IDs of manganese peroxidases (A) and lignin peroxidases (B) of *P. carnosa* and *P. chrysosporium* are obtained from *P. carnosa* v1.0 and *P. chrysosporium* v2.0. Alternative names are from MacDonald *et al*. [10] for *P. carnosa* and from Vanden Wymelenberg *et al*. [6] for *P. chrysosporium.***Figure S6.** Mycelial growth of *P. carnosa* and *P. chrysosporium* on heartwood and sapwood samples isolated from different hardwood and softwood species. Colony diameter was measured for *P. carnosa* grown on heartwood (A) and sapwood (B), and for *P. chrysosporium* grown on heartwood (C) and sapwood (D). Filled square (red), sugar maple; filled circle (green), yellow birch; filled triangle (blue), trembling aspen; filled diamond (pink), red spruce; open square (purple), white spruce; open circle (orange), balsam fir; open triangle (gray), red pine. Error bars show the standard deviation in biological triplicates. Since *P. chrysosporium* mycelia was no longer visible after day 8 of cultivation on heartwood of sugar maple, yellow birch, white spruce and balsam fir, those data were not obtained. **Figure S7.** Different modes of wood decay described by FT-IR analysis. (A) Grouping on Principal Components (PCs) 1 and 2 for normalized FT-IR data obtained from heartwood and sapwood samples of trembling aspen and red pine after cultivation of *P. carnosa* and *P. chrysosporium*. Circle; *P. carnosa*, triangle; *P. chrysosporium*, square; control (untreated wood samples). (B) PC loadings that distinguish wood samples treated with *P. carnosa* from corresponding control samples. For example, high positive loadings describe components in control samples that were lost in the decayed samples. Loadings for PC1 are shown for trembling aspen heartwood and red pine sapwood, while loadings for PC2 are shown for trembling aspen sapwood and red pine heartwood. (C) PC loadings that distinguish wood treated with *P. carnosa* from corresponding wood samples treated with *P. chrysosporium*. Horizontal dotted lines at magnitude |0.05| represent thresholds for loading significance. Corresponding wavenumbers (cm^-1^) are indicated for peaks with significant loadings; identities of significant wavenumbers are summarized in Additional file: Table. S17. Percent values given in y-axes denote the percent of total sample variance described by the PC. Click here for file

Additional file 4 **Table S7.** List of CAZy members computationally annotated in *P. carnosa*. List of protein IDs computationally annotated as CAZy members in *P. carnosa*. Click here for file

Additional file 5 **Table S10.** List of the genes involved in carbohydrate metabolism. List of *P. carnosa* and *P. chrysosporium* genes involved in carbohydrate metabolism accompanied with gene expression data of *P. carnosa*. Click here for file
