# Supplementary material for: Comparative genomics of the white-rot fungi, Phanerochaete carnosa and P. chrysosporium, to elucidate the genetic basis of the distinct wood types they colonize
Source: BMC Genomics. 2012 Sep 2;13:444. doi: 10.1186/1471-2164-13-444 (PMC3463431; doi:10.1186/1471-2164-13-444)
Supplement: Additional file 1 — Table S1.P. carnosa sequencing summary. This describes the summary of libraries that were constructed for genome sequencing. Table S2.P. carnosa assembly summary. This describes the summary of the genome sequence assembly. Table S3. Gene model support by different lines of evidence. Statistics of predicted gene models. Table S4. Summary of P.carnosa annotations. This summarizes genome annotation according to various classifications. Table S5. Top 50 PFAM domains in Phanerochaete genomes. List of top 50 PFAM domains annotated in Phanerochaete genomes. Table S8. Comparison of the number of CAZymes in wood decaying basidiomycotina. General comparison of CAZyme gene members in various basidiomycotina [5,6,12,17,78,79]. Table S9. CAZymes that showed <60% identity to Phchr orthologs. List and gene expression data of CAZy members showing low sequence identity with orthologs in P. chrysosporium[10]. Table S11. Comparison of the number of FOLymes in P. carnosa and selected Agaricomycotina. General comparison of FOLy members between several Agricomycotina [78]. Table S12. Summary of oxidoreductases potentially involved in lignocellulose degradation by P. carnosa (Phaca) and P. chrysosporium (Phchr). Summary and comparison of specific oxidoreductase members between Phanerochaetes. Table S13. Tandem duplication of P450 genes in basidiomycete genomes. Summary table of P450 tandem duplication in known basidiomycete genomes [5,6,12,17,78-80]. Table S14. P450ome classification in P. carnosa and its membership comparison with P. hrysosporium. Summary table of P450 members in P. carnosa and P. chrysosporium. Table S15. P450s upregulated in wood degrading cultures. List of P450 members in P. carnosa that were upregulated during wood degrading cultivation [10]. Table S16. UPLC peaks corresponding to wood-derived phenolic compounds that were transformed by P.carnosaor P. chrysosporium. Peak annotation and analysis of UPLC chromatograms. Table S17. Assignment of FT-IR peaks decomposed by [file 1471-2164-13-444-S1.pdf]

Supplemental tables

Table S1. *P. carnosa* sequencing summary.

Library	Library_Type	Raw_Reads	Raw_Bases	Trimmed_Bases	Assem_Reads	Assem_Bases	Coverage	Insert	Insert_StdDev
GCXT	454	3,250,968	1,314,960,768	1,313,748,704	3,127,914	1,260,244,475	29.05x	NA	
GUPU	454	1,000,756	405,503,493	404,997,892	974,429	396,177,643	9.13x	NA	
GHOC	454PE	1,312,895	327,864,098	295,946,764	1,234,416	287,653,280	6.63x	3560	889
GCNX	454PE	572,197	177,323,735	162,914,495	543,132	158,464,822	3.65x	14475	3618
GFYP	454PE	199,868	66,367,587	60,638,155	191,898	59,079,037	1.36x	6149	1537
GFYS	454PE	110,638	47,728,082	43,425,668	106,417	42,060,278	0.97x	5218	1729
GATS	SANGER	190,290	167,187,218	147,358,060	182,501	140,250,273	3.23x	2918	729
GUPW	SANGER	13,332	11,769,040	9,601,641	12,434	8,723,897	0.20x	39372	9843
NODE	SOLEXA	192,038	171,143,486	171,141,890	188,005	168,072,328	3.87x	NA	
**TOTAL**		6,842,982	2,689,847,507	2,609,773,269	6,561,146	2,520,726,033	58.10x		

Table S2. *P. carnosa* assembly summary.

Genome Assembly size	46.29Mb
Sequencing read coverage depth	58.10
# of contigs	2272
# of scaffolds	1137
Scaffold N50/ L50	6/3.53Mb

Table S3. Gene model support by different lines of evidence.

	P. carnosa	*P. chrysosporium*
# of models	13,937	10,048
% complete (start + stop)	12,449 (89%)	8352 (83%)
% supported by nr homolog	10,619 (76%)	ND
% supported by Swissprot homolog	7736 (56%)	6621 (66%)
% with Pfam domain	6449 (46%)	4769 (47%)
% with EST support (>25%)	12062(87%)	ND
% with full-length EST support	8823(63%)	ND
Gene length (nt)	1448	1363
Transcript length (nt)	1190	1116
Protein length (aa)	313	372
Exon length (nt)	159	138
Intron length (nt)	57	55
# exons / gene	4	5
# single exon genes	2695	1202

Table S4. Summary of *P.carnosa* annotations.

**KEGG pathway**		
Amino Acid Metabolism	594	4%
Biosynthesis of Polyketides and Nonribosomal Peptides	118	1%
Biosynthesis of Secondary Metabolites	375	3%
Carbohydrate Metabolism	588	4%
Energy Metabolism	134	1%
Glycan Biosynthesis and Metabolism	195	1%
Lipid Metabolism	568	4%
Metabolism of Cofactors and Vitamins	466	3%
Metabolism of Other Amino Acids	145	1%
Nucleotide Metabolism	268	2%
Biosynthetic pathways	402	3%
Xenobiotics Biodegradation and Metabolism	456	3%
**Total KEGG annotated genes**	4309	31%
**KOG Classification**		
Cell wall/membrane/envelope biogenesis	95	1%
Cell motility	5	0%
Posttranslational modification	700	5%
Signal transduction mechanisms	652	5%
Intracellular trafficking, screction, &vesicular transport	319	2%
Defense mechanisms	132	1%
Extracellular structures	69	0%
Nuclear structure	93	1%
Cytoskeleton	219	2%
RNA processing and modification	382	3%
Chromatin structure and dynamics	184	1%
Translation, ribosomal structure and biogenesis	346	2%
Transcription	396	3%
Replication, recombination and repair	231	2%
Energy production and conversion	393	3%
Cell cycle control, cell division, chromosome partitioning	219	2%
Amino acid transport and metabolism	252	2%
Nucleotide transport and metabolism	84	1%
Carbohydrate transport and metabolism	343	2%
Coenzyme transport and metabolism	101	1%
Lipid transport and metabolism	362	3%
Inorganic ion transport and metabolism	157	1%
Secondary metabolism	490	4%
**Total KOG annotated genes**	6224	45%
**GO Term**		
Biological process	3731	27%
Cellular component	1972	14%
Molecular function	5748	41%
**Total GO annotated genes**	6100*	44%*

*In GO, the same gene can be assigned to multiple categories.

Table S5. Top 50 PFAM domains in *Phanerochaete* genomes.

			Other 31 Basidiomycetes*			
**PfamId**	**Phchr**	**Phaca**	**Avg**	**Max**	**Min**	**Pfam Description**
PF00067	113	207	91	204	4	p450
PF07690	141	172	148	427	22	MFS_1
PF00400	109	202	113	197	49	WD40
PF00069	106	114	104	152	56	Pkinase
PF00106	84	77	82	189	14	adh_short
PF00646	70	86	74	199	7	F-box
PF00271	59	82	80	158	32	Helicase_C
PF00248	53	69	36	72	9	Aldo_ket_red
PF00026	52	68	24	51	2	Asp
PF01370	50	59	37	79	11	Epimerase
PF00076	50	55	56	79	19	RRM_1
PF00107	52	50	40	97	11	ADH_zinc_N
PF00005	50	48	38	64	17	ABC_tran
PF00501	30	64	25	76	8	AMP-binding
PF00172	35	56	68	244	5	Zn_clus
PF04082	36	52	45	198	2	Fungal_trans
PF05199	32	54	20	53	1	GMC_oxred_C
PF08240	47	38	40	112	12	ADH_N
PF00096	35	48	51	112	15	zf-C2H2
PF00004	39	44	37	49	17	AAA
PF02985	42	41	38	49	14	HEAT
PF00732	31	50	19	51	2	GMC_oxred_N
PF00098	57	23	40	166	1	zf-CCHC
PF01494	31	47	28	72	3	FAD_binding_3
PF00270	33	43	46	67	14	DEAD
PF01266	35	41	30	70	8	DAO
PF00385	69	6	6	24	1	Chromo
PF00083	39	36	42	123	9	Sugar_tr
PF00097	28	47	42	102	15	zf-C3HC4
PF00692	67	3	2	15	0	dUTPase
PF07993	29	39	27	68	7	NAD_binding_4
PF00561	27	39	26	56	8	Abhydrolase_1
PF00153	34	31	33	40	8	Mito_carr
PF08477	32	29	31	54	4	Miro
PF08242	29	31	28	68	9	Methyltransf_12
PF01753	23	37	27	129	1	zf-MYND
PF08241	29	30	31	60	11	Methyltransf_11
PF00071	30	28	30	53	6	Ras
PF07719	27	30	28	40	15	TPR_2
PF00734	28	28	8	44	0	CBM_1
PF00583	24	31	23	40	6	Acetyltransf_1
PF02798	16	37	16	56	2	GST_N
PF00023	20	32	29	92	12	Ank
PF08659	27	23	32	95	8	KR
PF00515	23	27	27	36	14	TPR_1
PF00226	22	27	29	50	13	DnaJ
PF00018	22	25	25	36	14	SH3_1
PF00702	25	22	25	38	8	Hydrolase
PF00149	19	27	22	31	13	Metallophos

*Gene counts include predicted gene models.

Table S8. Comparison of the number of CAZymes in wood-decaying basidiomycotina.

	GH	GT	PL	CE	Total no.	CBM
*Phanerochaete_carnosa*_HHB-10118-sp	193	74	8	17	292	43
Phanerochaete_chrysosporium [5, 6]	182	66	4	16	268	48
*Postia_placenta*_Mad-698-R [12]	143	75	6	10	234	16
*Serpula_lacrymans*_S7.3 [17]	154	63	6	12	235	29
*Schizophyllum_commune*_H4-8 [78]	236	75	16	30	357	30
Coprinopsis_cinerea [79]	211	72	13	51	347	89
Average	187	71	9	23	289	43

Table S9. CAZymes that showed <60% identity to Phchr orthologs.

Family	Protein	Identity	E-value	Putative target in wood component
GH5	183787	55.60%	1.00E-072	Cellulose/Hemicellulose
GH5	168586	55.56%	3.00E-130	Cellulose/Hemicellulose
GH5	253799	49.14%	3.00E-088	Cellulose/Hemicellulose
GH5	93346	44.07%	1.00E-020	Cellulose/Hemicellulose
GH5	194761	34.23%	8.00E-017	Cellulose/Hemicellulose
GH5	194783	30.63%	2.00E-010	Cellulose/Hemicellulose
GH7	191400	59.40%	1.00E-079	Cellulose
GH7	205265	55.71%	2.00E-079	Cellulose
GH7	258037	54.64%	6.00E-143	Cellulose
GH16	136813	58.68%	0.00E+000	Hemicellulose
GH16	127529	54.17%	2.00E-110	Hemicellulose
GH16	153747	33.51%	8.00E-019	Hemicellulose
GH23	258390	42.01%	0.38	
GH28	82797	28.32%	2.00E-013	Pectin
GH45	258456	37.66%	3.00E-010	Cellulose
GH61	251219	53.41%	1.00E-068	Cellulose
GH79	266539	57.41%	4.00E-032	Hemicellulose
GH79	265650	55.70%	2.00E-020	Hemicellulose
GH79	266185	53.01%	7.00E-020	Hemicellulose
GH79	189750	51.26%	4.00E-026	Hemicellulose
GH79	266219	50.00%	7.00E-023	Hemicellulose
GH79	257835	28.61%	4.00E-021	Hemicellulose
GH79	252537	24.53%	2.00E-018	Hemicellulose
GT1	210347	53.35%	3.00E-131	
GT1	205009	50.57%	2.00E-124	
GT1	205674	49.77%	1.00E-118	
GT1	205698	48.05%	2.00E-112	
GT22	180449	26.07%	1.00E-005	
GT22	214615	25.42%	3.00E-005	
GT22	160814	25.00%	0.38	
GT49	187889	50.00%	4.00E-022	
PL14	260328	37.50%	4.00E-044	
PL14	146440	37.16%	8.00E-032	
PL14	260353	30.89%	6.00E-006	
PL14	265869	29.91%	2.00E-006	
CE16	258413	50.00%	6.10E-001	Hemicellulose
CE16	255713	37.59%	8.00E-045	Hemicellulose
CE16	195373	28.43%	1.00E-023	Hemicellulose
CE16	185435	25.93%	1.90E-001	Hemicellulose
CE16	255913	25.28%	5.00E-017	Hemicellulose

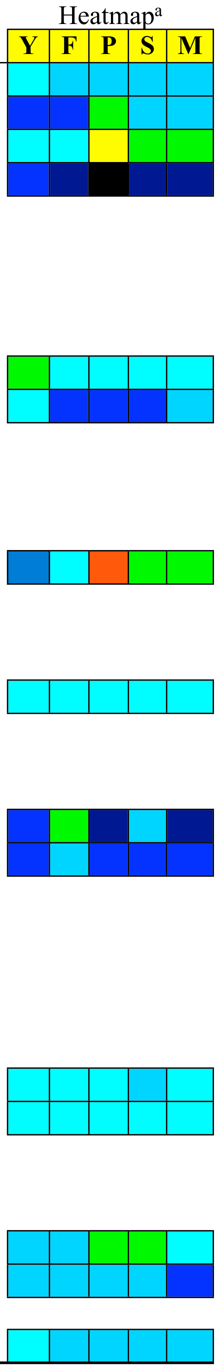

[a]Data obtained from MacDonald et al. [10]. Heat map represents the level of gene transcripts as described in Fig. S2

Table S11. Comparison of the number of FOLymes of *P. carnosa* and selected Agaricomycotina.

**Species**	**FOLyme**										
	LO1	LO2	LO3	LDA1	LDA2	LDA3	LDA4	LDA5	LDA6	LDA7	LDA8
Phanerochaete carnosa[a]	0	11	1	4	0	1	0	0	1	3	
Phanerochaete chrysosporium[a,b]	0	16	1	3	0	1	1	0	1	4	0
Coprinopsis cinerea[b]	17	1	1	18	0	0	0	0	1	2	0
Schizophyllum commune[b]	2	0	1	1	0	2	1	0	4	4	1
Postia placenta[b]	2	0	0	3	0	0	0	0	0	1	2
**Reference**[c]	LAC2_PLEOS	LIG8_PHACH	CDH_PHACH	AAC72747	VAOX_PENSI	AAA33747	P2OX_PHLGI	XP_959153	XP_002910108	AAD21025	AAB57849

Data from [a]this study; [b]Ohm et al. [78];

[C]Reference genes are Genbank Accessions of genes that have been characterized. *P. carnosa* sequences were annotated to the putative function of the reference protein when the bests hits to NCBI represented sequences of interest and the alignment showed at least 30% amino acid identity to the reference protein.

Table S12. Summary of oxidoreductases potentially involved in lignocellulose degradation by *P. carnosa* (Phaca) and *P. chrysosporium* (Phchr).

Putative Function	EC Class	Phaca (no.)	Phaca ID	Phchr (no.)	Phchr ID	Ref. Gene
**Peroxide generation**						
Methanol oxidase[a]	1.1.3.13	4	252324, 121157, 126707, 213078	3	126879, 5574, 6010	ALOX_PICAN
Aryl alcohol oxidase[a]	1.1.3.7	4	260543, 100299, 132559, 147295	3	37188, 135972, 6199	AAC72747
Glucose oxidase[a]	1.1.3.4	0 / 1	- / 179599	0 / 1	- / 6270	GOX_ASPNG / XP_002910108
Pyranose-2-oxidase	1.1.3.10	0	-	1	137275	
Glyoxal oxidase	-	1	258261	1	11088	
Copper radical oxidase	-	5	123913, 259359, 143144, 263533, 263528	6	259359, 123913, 258261, 8882, 121730, 121818	
**Iron reduction and homeostasis**						
Quinone reductase[a]	1.6.5.5	3	254412, 114036, 141788	4	121028, 129887, 10307, 139901	AF465406
Glycoprotein iron reductase	-	3	249086, 169427, 258034	2	AB236889 AB236890	
Cellobiose dehydrogenase	1.1.99.18	1	259608	1	11098	
Iron ferroxidase	1.16.3.1	1	141262	1	26890	
**Lignin modification**						
Lignin peroxidase	1.11.1.13	4	263501, 213241, 212237, 152156	10	10957, 121822, 131738, 6811, 11110, 122202, 8895, 121806, 131707, 131709	

Mn peroxidase	1.11.1.16	7	262882, 256980, 94399, 256984, 256997, 256991, 144982	5	140708, 3589, 878, 8191, 4636
Low redox peroxidase	1.11.1.7				
Chloroperoxidase[b]	1.11.1.10	3	263009, 249438, 254678	1	1710
Laccase	1.10.3.2	0	-	0	-

[a]best hit and ≥30% amino acid identity with reference gene; [b]contains chloroperoxidase Interpro domain.

Reference genes are Genbank Accessions of genes that have been characterized. *P. carnosa* sequences were annotated to the putative function of the reference protein when the bests hits to NCBI represented sequences of interest and the alignment showed at least 30% amino acid identity to the reference protein.

Table S13. Tandem duplication of P450 genes in basidiomycete genomes.

Genome origin	P450 genes	Tandemly duplicated genes	Tandem duplication sites	Duplicated genes (%)
*Phanerochaete_carnosa*_HHB-10118-sp	244	80	38	33
*Phanerochaete_chrysosporium*_RP-78 [5, 6]	131[a]	23	15	18
Postia placenta MAD-698 [12]	263	59	24	22
*Serpula_lacrymans*_S 7.3 [17]	136	37	16	27
*Schizophyllum_commune*_H4-8 [78]	106	31	14	29
*Coprinopsis_cinerea*_Okayama 7 [79]	122	45	16	37
Laccaria bicolor S238N-H70 [80]	69	23	15	33

[a]Number of genes which has unique protein ID in *P. chrysosporium* v2.0 database.

Table S14: P450ome classification in *P. carnosa* and its membership comparison with *P. chrysosporium*.

Clan	Family	Sub-family	*P. chrysosporium* No. of P450s		*P. carnosa* No. of P450s	
			Family	Clan	Family	Clan
CYP51	CYP51	F	1	**4**	2	**5**
	CYP5025	NS			1	
	CYP5156	A	2		1	
	CYP5157	A	1		1	
CYP52	CYP63	A	4	**24**	7	**32**
		B	1		1	
		C	2		1	
	CYP5137	A	2		4	
	CYP5141	A	4		6	
		B	1			
		C	1		1	
		D	1		1	
		NS			1	
	CYP5142	A	3		1	
		B	1		2	
		C	1		4	
		D	1			
		E	1		1	
	CYP5151	A	1		1	
		B			1	
CYP53	CYP53	C	1	**4**	6	**10**
		NS			1	
	CYP5140	A	1		1	
	CYP5143	A	2		2	
CYP54	CYP512	B	5	**14**	2	**27**
		C	2		9	
		D	1			
		E	1		1	
		F	1		2	
		G	2		7	
		H	1		5	
		J	1			
		NS			1	
CYP61	CYP61	A	1	**1**	1	**1**
CYP64	CYP502	B	1	**61**	1	**124**
	CYP620	NS			1	
	CYP5037	A	1		1	
		B	3		5	
		C	1		1	
		NS			1	
	CYP5144	A	15		19	
		B	1		7	
		C	8		16	
		D	5		3	
		E	1		1	
		F	1		5	
		G	1		7	
		H	1		12	
		J	1		1	
	CYP5145	A	3		2	
	CYP5146	A	4		5	

		B	1		1	
		C	1		9	
	CYP5147	A	4		6	
		B	1		1	
		C	1			
	CYP5148	A	2		3	
		B			4	
	CYP5149	A	1		3	
	CYP5152	A	2		4	
	CYP5158	A	1		5	
CYP67	CYP5035	A	7	**18**	6	**22**
		B	3		3	
		C	1		5	
		D	1			
		E	1			
	CYP5036	A	3		3	
		B	1			
		C	1		5	
CYP505	CYP505	D	7	**7**	4	**4**
CYP534	CYP5138	A	1	**11**	2	**29**
	CYP5139	A	1		11	
	CYP5150	A	5		6	
		B	1		4	
		C	1			
	CYP5153	A			1	
	CYP5154	A	1		3	
		NS			1	
	CYP5155	A	1		1	
CYP547	CYP5136	A	5	**5**	6	**8**
		NS (2)			2	
UA	CYP6001	C			1	**4**
		NS (3)			3	
**Authentic P450s**			**149**			**266**
Tentative P450s			-			5
Pseudo/doubtful P450s			10			13
Non P450			-			1
**Total Hits**			**159**			**285**

An initial search in the machine annotated *P. carnosa* genome revealed 285 P450 hits. These sequences were subjected to manual annotation based on the presence of the conserved P450 characteristic domains, including the oxygen-binding motif and the heme-binding domain. Initially, 239 gene models that contained both the conserved domains were considered as “Authentic P450s”. One sequence showed no convincing homology to P450 proteins and was therefore grouped as “Non-P450”. Further analysis of the remaining 45 P450 sequences revealed the characteristic P450 signature motifs in 27 gene models which were then grouped under the list of authentic P450s. Taken together, the total authentic P450 count for *P. carnosa* came up to 266. Of the remaining 18 of the 45 sequences, 5 sequences were very short and their flanking genomic DNA sequence quality was not as good (due to errors in genome sequencing) and thus it was not possible to predict the P450 signature motifs. However, the available protein sequences of these 5 P450s showed high homology (>75-83%) to the known P450s; hence we grouped them as “tentative P450s”. The remaining 13 sequences showed either one or no P450 characteristic domains in the existing gene models as well as in manually predicted gene models. These 13 P450s were therefore grouped as “Doubtful P450s or Pseudogenes”. Abbreviations: NS, New-subfamily; UA, unassigned clan.

Table S15. P450s upregulated in wood degrading cultures.

Protein ID	Clan	Family	Heatmap				
			Y	F	P	S	M
260638	CYP547	CYP5136					
259665	CYP52	CYP5137					
183109	CYP53	CYP53					
256887	CYP64	CYP5152					
254221	CYP64	CYP5148					
206285	CYP54	CYP512					
265475	CYP64	CYP5152					
164255	CYP64	CYP5158					
246674	CYP54	CYP512					
194437	CYP64	CYP5148					
109856	CYP52	CYP63					
262256	CYP547	CYP5136					
265429	CYP534	CYP5150					
253633	CYP534	CYP5150					
249950	CYP534	CYP5150					
212559	CYP53	CYP53					
211048	CYP52	CYP5137					
263271	CYP52	CYP5141					
211944	CYP52	CYP5141					
257741	CYP67	CYP5036					
261326	CYP52	CYP5141					
208499	CYP64	CYP5144					
250554	CYP534	CYP5139					
265238	CYP64	CYP5144					
209385	CYP64	CYP5144					
256807	CYP64	CYP5144					
253615	CYP64	CYP5037					
207382	CYP64	CYP5144					

Gene expression data were obtained from MacDonald et al. [10]. Y: YMPG culture, F: balsam fir, P: lodgepole pine, S: white spruce, M: sugar maple. Heat map represents the level of gene transcripts as described in Fig. S2.

Table S16. UPLC peaks corresponding to wood-derived phenolic compounds that were transformed by *P. carnosa* or *P. chrysosporium*.

Retension time	Component	S. maple		Y. birch		T. aspen		R. spruce		W. spruce		B. fir		R. pine	
		Pca	Pch	Pca	Pch	Pca	Pch	Pca	Pch	Pca	Pch	Pca	Pch	Pca	Pch
**Heartwood, 350nm**															
1.81	ρ-Coumaric acid									+					
2.49	(-) – Epicatechin gallate									++	++		+		
2.88	Benzoic acid	++	++			++	++	++	++			+	+	+	
3.35	Kaempferol 3-O-β-D-glucoside							++	++	++					
3.84	Naringin									++					
4.89	Kaempferol 3-O-β-L-rhamnoside		+												
5.31	Unidentified		+												
6.77	Unidentified		+												
6.93	Unidentified	+	+							+					
8.32	Unidentified							++	++	++					
9.24	Unidentified						+								
**Heartwood, 280nm**															
7.91	Unidentified									++	+				
**Sapwood, 350nm**															
2.89	Benzoic acid													++	+
3.5	Quercetin 3-O-β-L-rhamnoside													++	+
7.29	Luteolin													+	
9.08	Unidentified	++	+												
9.11	Kaempferol	++	+												
9.27	Unidentified	++	+												
9.38	Unidentified	++	+												
9.53	Unidentified	++	+												
9.67	Unidentified	++	+												
**Sapwood, 280nm**															
1.62	Unidentified									+					

++: Significantly decreased, +: Detectable decrease, Blank: Not significantly affected or peaks not observed.

Table S17. Assignment of FT-IR peaks decomposed by *P. carnosa* and *P. chrysosporium.*

		PCA loading			
Wave number (cm-1)	Peak description	Aspen H	Aspen S	Pine H	Pine S
Peaks decreased by P. carnosa					
1700-1550	Conjugated carbonyl stretches, conjugated and nonconjugated C=C stretches; characteristic of both lignin and carbohydrates [81]				1622*
1515–1505	C1⁄4C stretching of the aromatic ring (G units)		1522<br>1508		1518
1470–1455	C–H asymmetric deformation in –OCH3, CH2 in pyran ring symmetric scissoring		1465		
1375–1365	CH bending in cellulose I and cellulose II and hemicellulose			1384	1384*
1268-1273	Guaiacyl ring breathing with carbonyl stretching [82]		1261		
1235–1230	Syringyl ring breathing and C–O stretching in lignin and xylan		1229		
1162–1125	C–O–C asymmetric stretching in cellulose I and cellulose II		1127		
1128–1110	Aromatic C–H in-plane deformation (typical for S units), C1⁄4O stretch	1124*	1127		
1128–1110	Aromatic C–H in-plane deformation (typical for S units), C1⁄4O stretch			1105	
1086–1075	C–O deformation in secondary alcohols and aliphatic ethers			1091	
1060–1015	C–O valence vibration mainly from C(3)–O (3)H			1062<br>1035	1056<br>1030
1047–1004	C–O stretching in cellulose I and cellulose II			1035	1030
996–985	C–O valence vibration	990*			
Additional peaks decreased only by P. chrysosporium					
1650–1640	Water associated with lignin or cellulose	1644			
1470–1455	C–H asymmetric deformation in –OCH3, CH2 in pyran ring symmetric scissoring				1477
1162–1125	C–O–C asymmetric stretching in cellulose I and cellulose II			1168	1183
1128–1110	Aromatic C–H in-plane deformation (typical for S units), C1⁄4O stretch			1125<br>1114	
1060–1015	C–O valence vibration mainly from C(3)–O (3)H		1060<br>1037		
1047–1004	C–O stretching in cellulose I and cellulose II			1003	
996–985	C–O valence vibration			985	981

*Peaks representing components decomposed only by *P. carnosa* as shown in Fig. S7(C).
Peak description not referenced in the table are from Popescu et al. [83]

Table S18. Gradient method of UPLC elution.

Time	Flow Rate (mL/min)	% A	% B	Curve
Initial	0.50	95.0	5.0	0
1.00	0.50	85.0	15.0	6
8.00	0.50	80.0	20.0	6
10.00	0.60	50.0	50.0	6
12.00	0.60	20.0	80.0	6
13.00	0.60	95.0	5.0	6

Solvent A: 0.1% Formic Acid

Solvent B: Acetonitrile
