# Supplementary material for: Comparative genomics of the white-rot fungi, Phanerochaete carnosa and P. chrysosporium, to elucidate the genetic basis of the distinct wood types they colonize
Source: BMC Genomics. 2012 Sep 2;13:444. doi: 10.1186/1471-2164-13-444 (PMC3463431; doi:10.1186/1471-2164-13-444)
Supplement: Additional file 3 — Figure S1. Pictures of P. carnosa and P. chrysosporium grown on various carbon sources. P. carnosa grew significantly slower than P. chrysosporium; as a result, pictures were taken after 13 and 3 days of cultivation for P. carnosa and P. chrysosporium, respectively. Cultivations were performed in duplicate and no significant differences in colony diameter or thickness were observed between the duplicates on any of the carbon sources. Monomeric and oligomeric carbon sources were used at a final concentration of 25 mM, while pure polymers were used at a final concentration of 1%. Crude plant biomass was used at a final concentration of 3%. Relative growth was determined by comparing the radius and density of the mycelia on a particular carbon source to that on D-glucose. The extent of growth relative to plates containing glucose are summarized as follows, from high to non-detectable: +++, ++, +, ±, -. This semi-quantitative, consistent assessment of both colony diameter and thickness allowed comparisons to include a broad range of substrates, including those that yield too little mycelia for accurate weight measurement, or that could interfere with protein measurements or ergosterol production [84,85]. Complete growth profiles of P. carnosa and P. chrysosporium, and other fungi can be found at [19]. Figure S2. Phylogenetic tree of GH61 enzymes from P. carnosa and P. chrysosporium. Proteins are labeled with protein IDs from the JGI databases for Phanerochaete carnosa v1.0 (Phaca) and Phanerochaete chrysosporium v2.0 (Phchr). The sequences were aligned using MAFFT, and the tree was drawn by FigTree. In the heatmap bar, abbreviations are; Y, YMPG; F, balsam fir; P, lodgepole pine; S, white spruce; M, sugar maple [10]. Heat map represents the number of sequence reads per million kb as shown in this figure and described in [10], 0 (black) to 50,000 (pink). Figure S3. Phylogenetic tree of GH5 enzymes in P. carnosa and P. chrysosporium. The tree was generated as described in [file 1471-2164-13-444-S3.pdf]

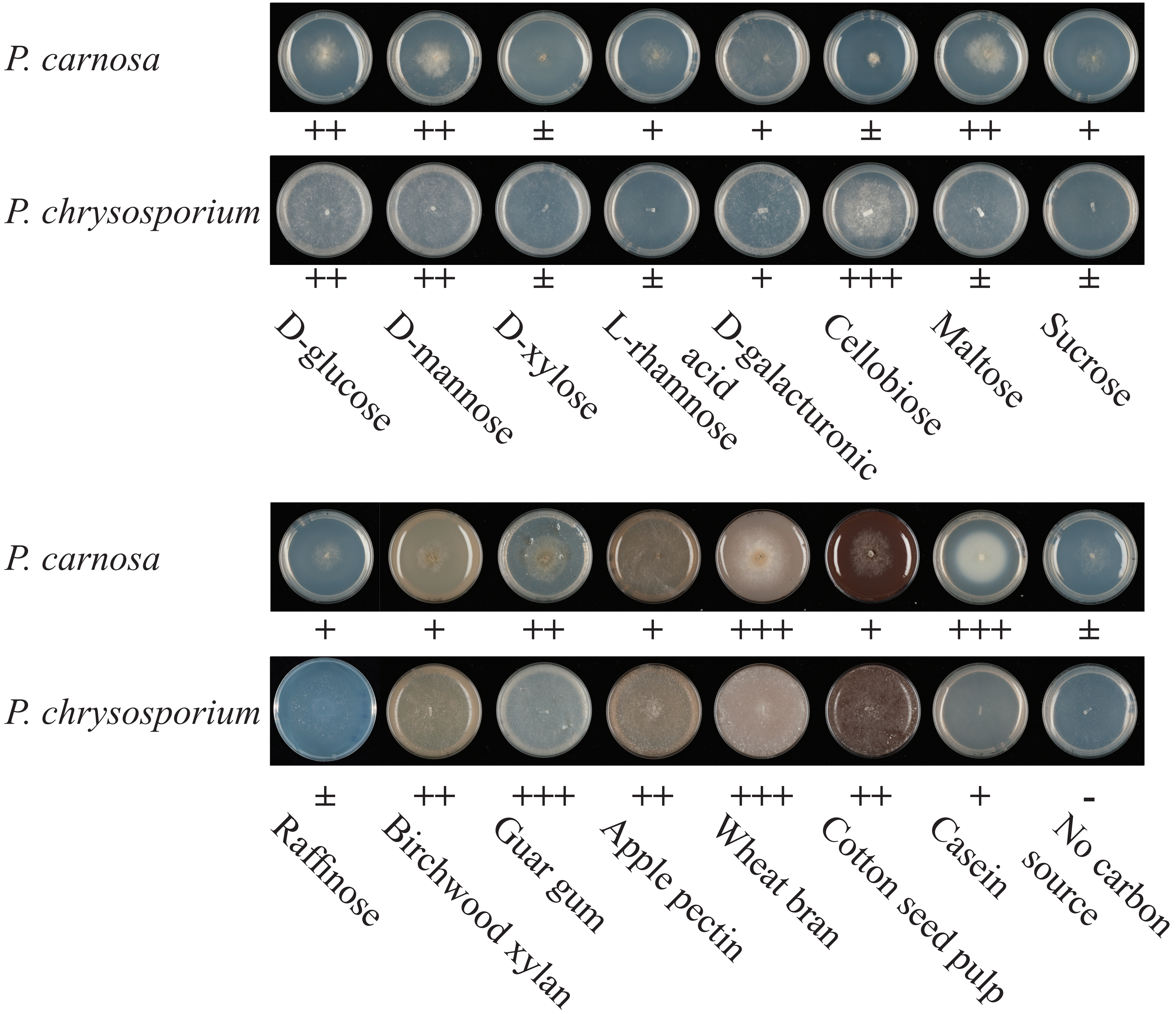

Fig. S1. Pictures of *P. carnosa* and *P. chrysosporium* grown on various carbon sources.

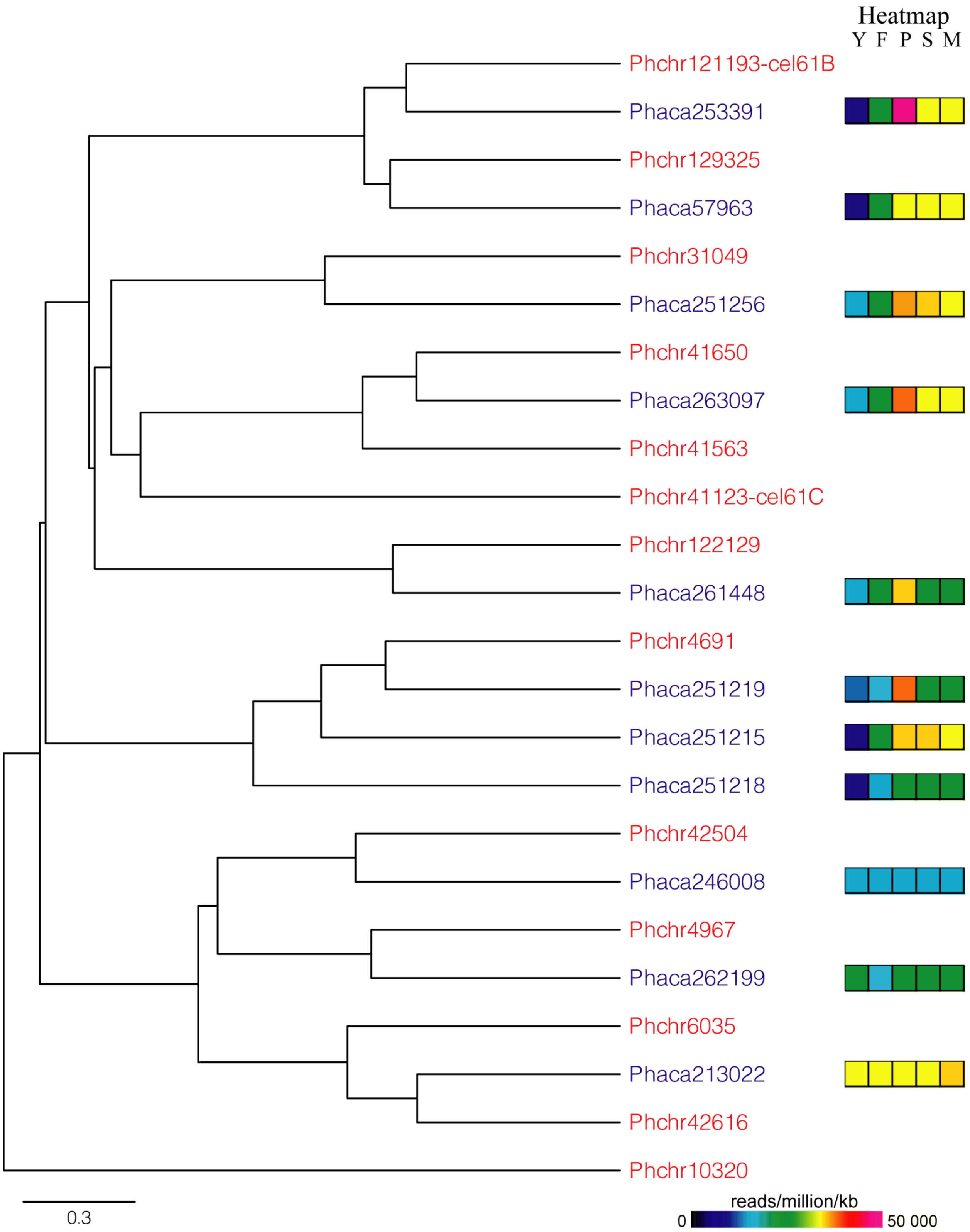

Fig. S2. Phylogenetic tree of GH61 enzymes from *P. carnosa* and *P. chrysosporium*.

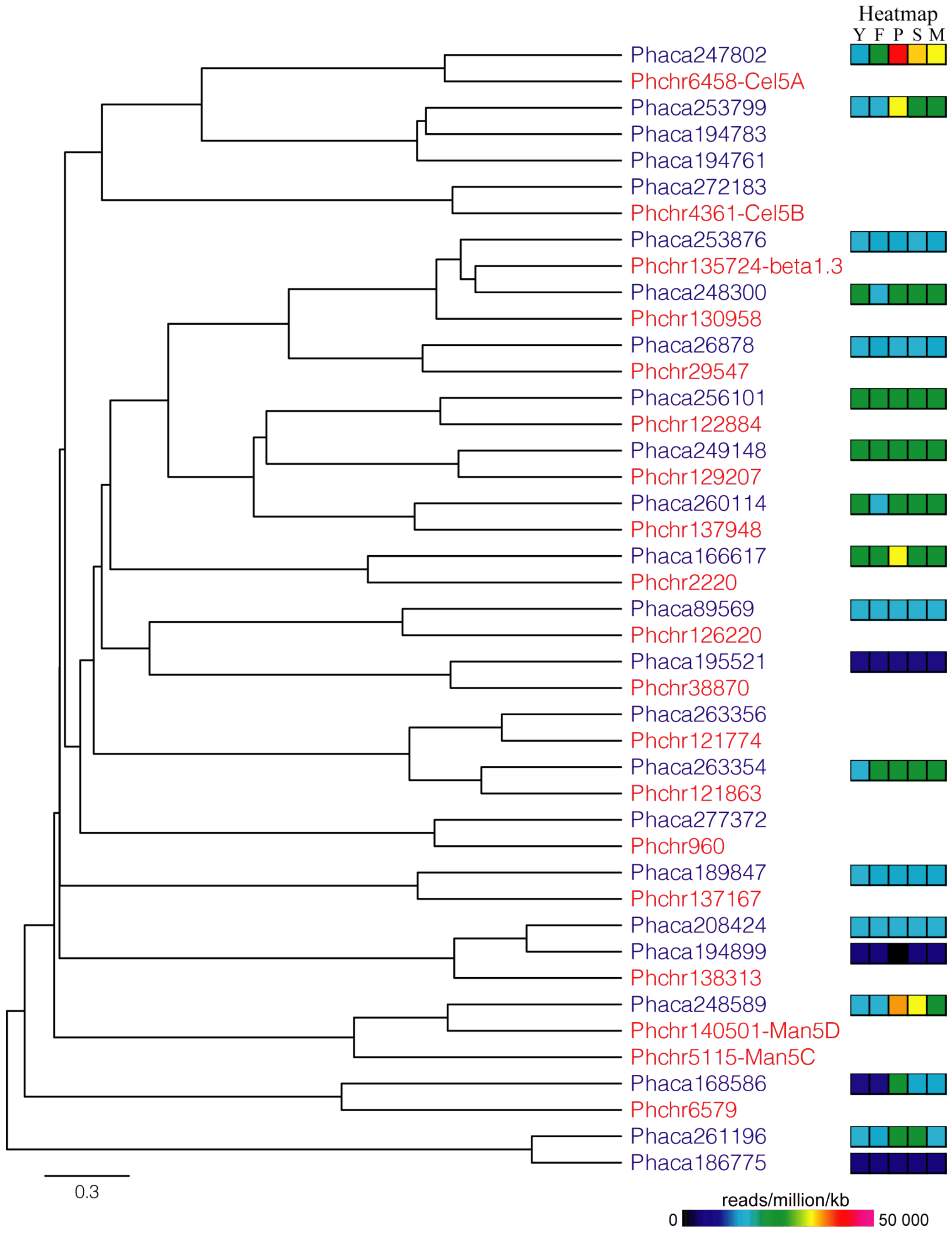

Fig. S3. Phylogenetic tree of GH5 enzymes in *P. carnosa* and *P. chrysosporium*.

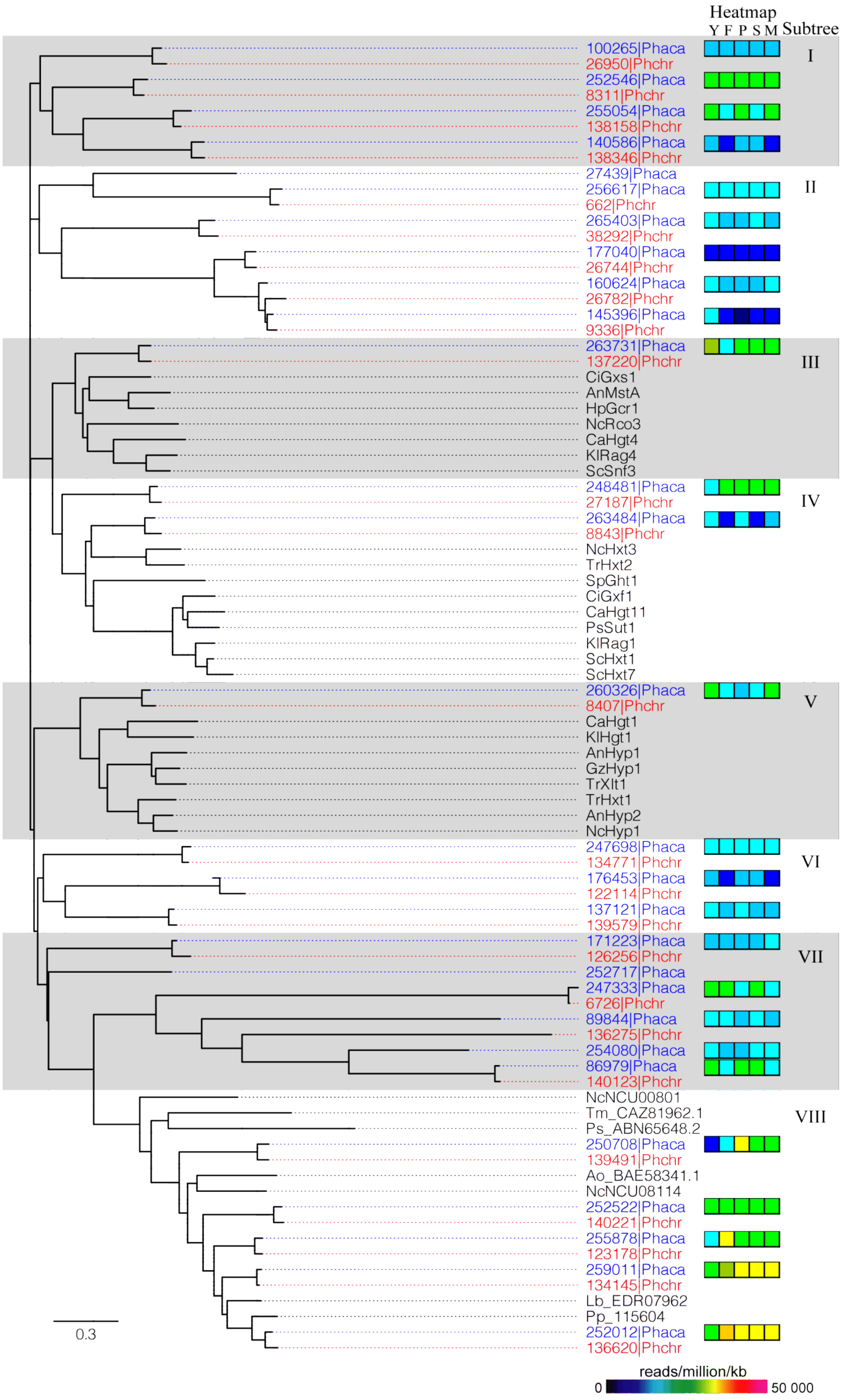

Fig. S4. Phylogenetic tree of predicted sugar transporters and permeases from genomes of *P. carnosa* and *P. chrysosporium.*

A

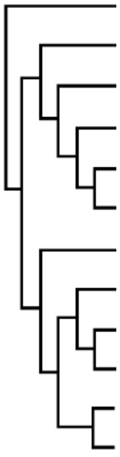

Species	Protein ID	Position	Introns	Alternative Name*
P. carnosa	144982	5:3175629-3177912	1-2-3-4-5-6-7	1138
P. carnosa	256991	5:3189583-3192656	1- 3-4-5-6-7	31
P. carnosa	256980	5:3167371-3169157	1- 3-4-5-6-7	383+789
P. carnosa	94399	5:3208808-3210366	1-2-3-4-5-6-7	1168
P. carnosa	256997	5:3195944-3197614	1-2-3-4-5-6-7	1579
P. carnosa	256984	5:3180144-3181864	1-2-3-4-5-6-7	697
P. carnosa	262882	10:495184-496977	1-2- 4-5-6-7	45
P. chrysosporium	878	1:2814195-2815673	1- 3-4-5-6-7	*mnp3*
P. chrysosporium	140708	15:846535-848049	1-2- 4-5-6-7	*mnp1*
P. chrysosporium	8191	15:853637-855322	1-2- 4-5-6-7	*mnp4*
P. chrysosporium	3589	5:505970-507645	1-2-3-4-5-6-7	*mnp2*
P. chrysosporium	4636	7:1395073-1396542	1-2-3- 5-6-7	*mnp5*

B

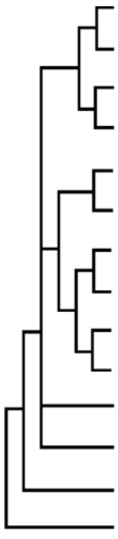

Species	Protein ID	Position	Introns	Alternative Name*
P. carnosa	263501	10:1969940-1971733	1-2-3-4-5-6- 8-9-10	9
P. carnosa	213241	10:1976572-1979063	1-2-3-4-5-6- 8-9-10	489
P. carnosa	152156	10:2018444-2020054	1-2-3-4-5-6-7-8-9-10	8106
P. carnosa	212237	9:404873-406916	2-3-4-5-6-7-8-9-10	9982+9983
P. chrysosporium	10957	19:373851-375408	1-2-3-4-5-6- 9-10	*lipA*
P. chrysosporium	131707	19:451627-454927	1-2-3-4-5-6- 9-10	*lipI*
P. chrysosporium	121822	19:376752-378294	1-2-3-4-5-6- 9-10	*lipB*
P. chrysosporium	11110	19:358710-360433	1-2-3-4-5-6- 9-10	*lipE*
P. chrysosporium	8895	19:448641-450192	1-2-3-4-5-6- 9-10	*lipG*
P. chrysosporium	121806	19:441862-443404	1-2-3-4-5-6- 9-10	*lipH*
P. chrysosporium	131738	19:393154-394751	1-2-3-4-5-6-7- 9-10	*lipC*
P. chrysosporium	122202	9:1444920-1446516	1-2-3-4-5-6- 8-9-10	*lipF*
P. chrysosporium	131709	19:439914-441504	1-2-3-4-5-6-7-8- 10	*lipJ*
P. chrysosporium	6811	11:1416506-1418221	2-3- 5-6-7-8-9-10	*lipD*

Fig. S5. Phylogeny, genome position, and intron distribution of genes encoding manganese peroxidases and lignin peroxidases.

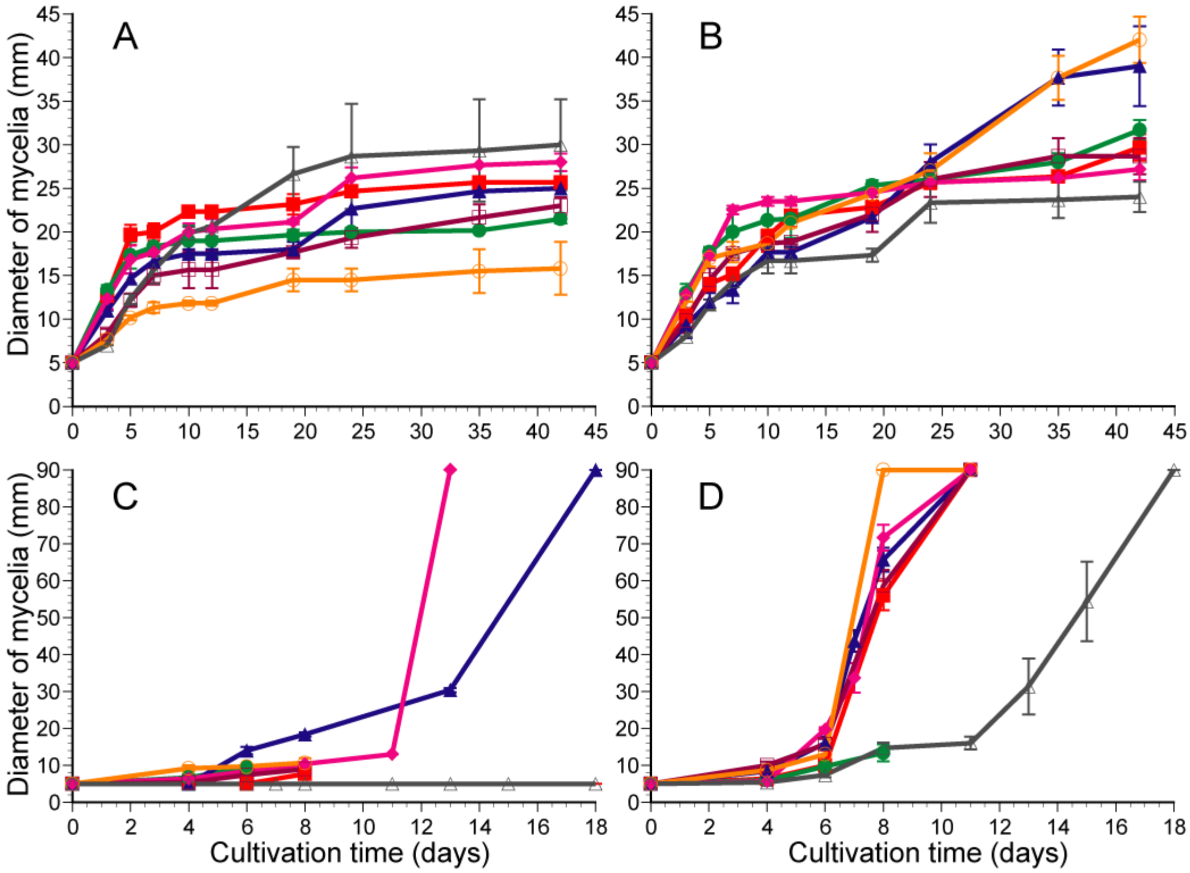

Fig. S6. Mycelial growth of *P. carnosa* and *P. chrysosporium* on different hardwood and softwood species.

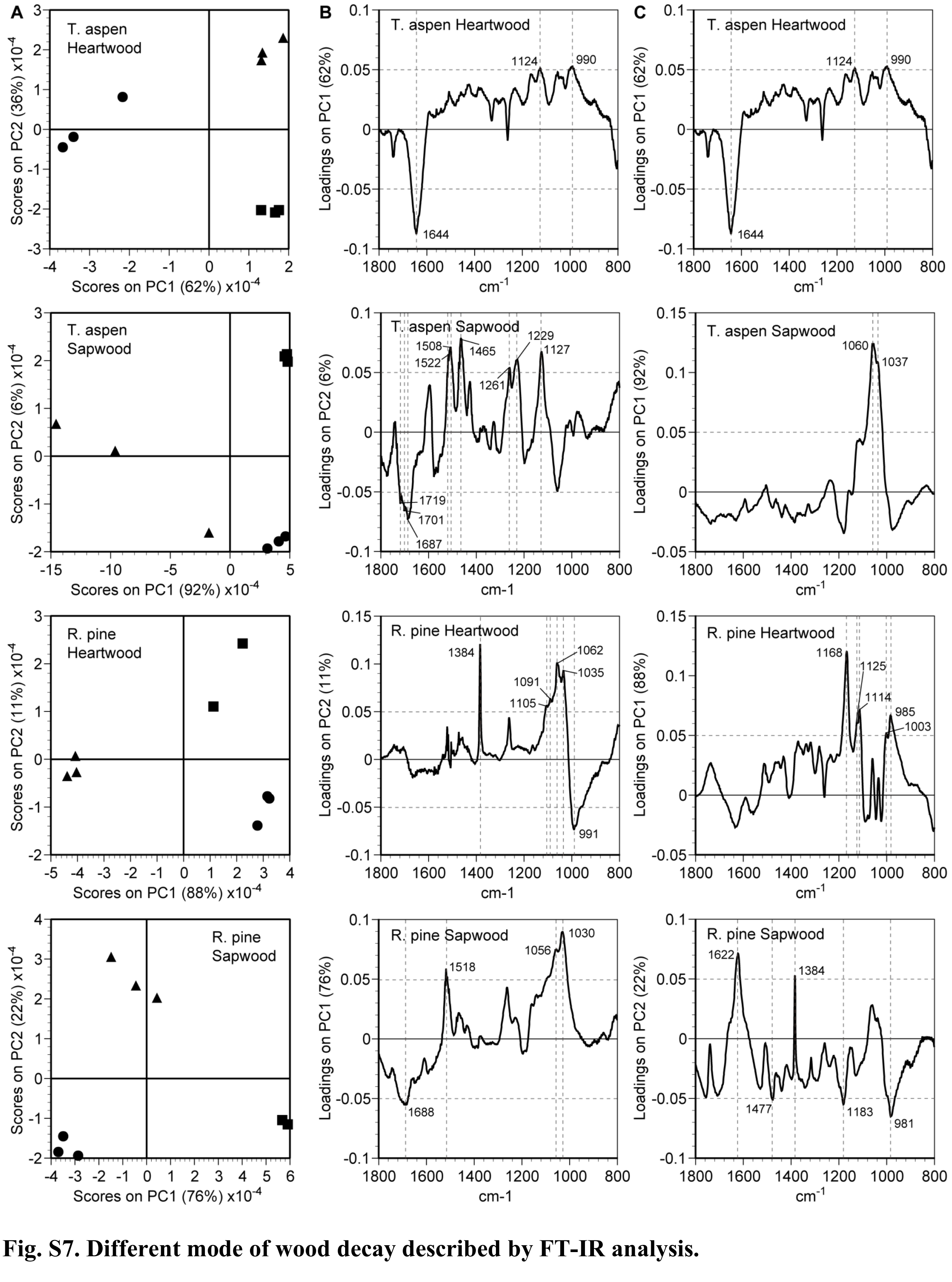

Fig. S7. Different mode of wood decay described by FT-IR analysis.
